# Cell-type-specific optogenetic stimulation of the locus coeruleus induces slow-onset potentiation and enhances everyday memory in rats

**DOI:** 10.1073/pnas.2307275120

**Published:** 2023-11-06

**Authors:** Dorothy Tse, Lucia Privitera, Anna C. Norton, Francesco Gobbo, Patrick Spooner, Tomonori Takeuchi, Stephen J. Martin, Richard G. M. Morris

**Affiliations:** ^a^Centre for Discovery Brain Sciences, Edinburgh Neuroscience, University of Edinburgh, Edinburgh EH8 9JZ, United Kingdom; ^b^Department of Psychology, Edge Hill University, Omskirk L39 4QP, United Kingdom; ^c^School of Systems Medicine, University of Dundee, Dundee DD1 4HN, United Kingdom; ^d^Barts and the London School of Medicine, Institute of Health Sciences Education, Queen Mary University of London Malta Campus, Victoria VCT 2570, Malta; ^e^Danish Research Institute of Translational Neuroscience, Nordic-European Molecular Biology Laboratory Partnership for Molecular Medicine, Aarhus University, Aarhus 8000, Denmark; ^f^Center for Proteins in Memory, Danish National Research Foundation, Department of Biomedicine, Aarhus University, Aarhus 8000, Denmark

**Keywords:** memory, long term potentiation, synaptic tagging and capture, locus coeruleus, dopamine

## Abstract

Memory formation is typically divided into phases associated with encoding, storage, consolidation, and retrieval. The neural determinants of these phases are thought to differ. This study first investigated the impact of the experience of novelty in rats incurred at a different time, before or after, the precise moment of memory encoding. Memory retention was enhanced. Optogenetic activation of the locus coeruleus mimicked this enhancement induced by novelty, both when given before and after the moment of encoding. Optogenetic activation of the locus coeruleus also induced a slow-onset potentiation of field potentials in area CA1 of the hippocampus evoked by CA3 stimulation. Despite the locus coeruleus being considered a primarily noradrenergic area, both effects of such stimulation were blocked by the dopamine D1/D5 receptor antagonist SCH 23390. These findings substantiate and enrich the evidence implicating the locus coeruleus in cellular aspects of memory consolidation in hippocampus.

## Significance

Other events happening around the time a memory is encoded can modulate how well such a memory is retained. The significance of this study is in revealing that environmental novelty either before or after memory encoding can enhance the retention of allocentric spatial memory, and this can be mimicked by optogenetic light activation of a brain area involved in neural activity in response to novelty. This intriguing perievent neural modulation is also associated with an optogenetically induced slow-onset potentiation of synaptic strength in the hippocampus which likely contributes to the enhancement of memory retention.

A key issue in the neurobiology of memory is the selectivity of forgetting. How is it that we remember some things but not others? Such selectivity is a positive feature of memory because we are protected from remembering every detail of daily events for long periods of time but can retain only what matters. Determinants of memory retention include both features at exactly the moment of memory encoding, such as effective attention or characteristics of the information being remembered (e.g., salience, novelty, emotional significance) (1, 2), and independent events happening around the same time. Both can influence the temporal persistence of memory over time. One determinant of this is the novelty of other unrelated events which can cause a penumbra of enhanced retention. An example is “flashbulb memory” in which a surprising and/or shocking event is not only itself remembered but also causes an enhanced penumbra of retention of other proximal events that may be relatively innocuous and would ordinarily be forgotten quickly (3). Memories of events surrounding the tragedy of 9/11 are of this character, although the enhanced incidental memories of that day have been shown to be not always without error (4). We suspect that novelty-associated enhancement of memory is a routine facet of everyday memory and not only something that happens on rare occasions. Surprising children during class can be used by teachers in school to enhance the memory of specific items (5). We here further develop our investigations of this phenomenon in an animal model.

Guided by the synaptic tagging and capture (STC) hypothesis of cellular consolidation (6, 7), in vitro electrophysiological studies have established that strong afferent tetanic stimulation of glutamatergic and neuromodulatory inputs to the CA1 region of the hippocampus not only produces a long-lasting, protein synthesis–dependent, long-term potentiation (LTP) in the stimulated pathway but can also enable a weak decaying LTP to last longer when it is induced in an independent pathway targeting the same population of neurons (6–8). This phenomenon has been observed at the level of single synapses (9) and in vivo (10). It is generally explained in terms of the local capture of plasticity-related proteins (PRPs) that are synthesized in response to the activation of neuromodulatory afferents (7, 8, 11, 12). The STC concept has been extended to include the phenomenon of “behavioral tagging” in which a surprising event (e.g., novelty exploration) has been shown to enhance the retention of weakly encoded memory traces which ordinarily decay within a day (13, 14).

In a study seeking to mimic the impact of novelty through direct optogenetic activation of a neuromodulatory catecholaminergic pathway from the locus coeruleus (LC) to the hippocampus in mice, the encoding of a weak decaying memory showed enhanced retention over time (15). The use of an optogenetic strategy is not without conceptual problems (16), but a gain-of-function approach can shed light on causal mechanisms. The LC was chosen for investigation because, in the same study, optetrode recordings from this nucleus revealed a striking increase in activity of LC-tyrosine hydroxylase-expressing (LC-TH^+^) neurons during novelty exploration. Such “perievent” novelty (i.e., the novelty of events other than the one being remembered but occurring around the same time) might contribute to enhanced retention more generally. One plausible mechanism posits that the induction of novelty elicits the activation of neuromodulatory neurons, triggering the release of a catecholaminergic transmitter within the hippocampus during the period of glutamatergic-mediated memory encoding. This concurrent activation subsequently engages signal transduction pathways that facilitate the synthesis and availability of PRPs, their localization at synapses marked for capture, thereby culminating in synaptic stabilization (8).

These findings contribute to a body of work implicating activity in the LC in various aspects of cognition, memory formation, and retention (17, 18). In addition to diverse consequences that likely include triggering PRP synthesis, it could also have a direct effect on neuronal excitability that influences the subsequent allocation of memory traces at specific neurons at the time of encoding (19, 20). In contrast, the STC model is symmetric regarding the synergistic effect of neuromodulation before or after encoding. However, when neuromodulation happens first, not only may there be enhanced excitability, but additional mechanisms may come into play. Navakkode et al. (21) showed in vitro that cooccurrent glutamatergic and dopaminergic activation of the CA1 region of the hippocampus with the dopamine D1/D5 receptor agonist SKF 38393 causes a slow-onset potentiation (SOP) of glutamatergic synaptic transmission over 30 to 60 min with a circa 15 to 25% increase in the magnitude of field potentials. We became interested in this SOP phenomenon as our optogenetic study had replicated this finding in mice in vitro but we had studied it only over a short timescale (15).

A last and important facet of the experimental design was our discovery that the event arena protocols used in our earlier studies of the retention of recent everyday memories are ambiguous with respect to the frame of reference of memory encoding, specifically regarding whether animals employ an allocentric or egocentric strategy to execute the task (22). An egocentric framework involves the incorporation of spatial information regarding an individual’s position within the environment, while the allocentric framework entails the integration of spatial information pertaining to the positional relationships among objects. To establish a robust protocol enabling the acquisition of allocentric memory representation within the event arena, analogous to the human capacity to recall the specific location of daily events, we implemented a strategic modification to our recency everyday memory task. This involves the introduction of an allocentrically defined “home-base” within the arena. The incorporation of this allocentric reference point aimed to facilitate the encoding of memories from an observer-independent perspective. The rats were required to find their reward in the arena and then carry it to this home-base ensuring that only allocentric recency memory formation happens (23). This occurs in the hippocampus downstream of egocentric memory processing in afferent cortical regions (24–26).

Behavioral and electrophysiological studies in vivo were used to determine whether 1) the experience of either novelty or optogenetic activation of LC-TH^+^ neurons, before or after memory encoding, would enhance the retention of allocentrically coded spatial memory over 24 h (i.e., beyond the usual forgetting period of a few hours); 2) activation of LC-TH^+^ neurons would also cause SOP in vivo measured in the CA1 region of the hippocampus, and 3) comparable pharmacological sensitivity would be observed across the behavioral and electrophysiological studies. Male transgenic rats expressing Cre recombinase in TH^+^ neurons (Th-Cre) (27) were used as subjects in which a Cre-inducible ChrimsonR construct had been virally transfected into the LC together with intracerebral light guides for optogenetic activation in vivo. Intracerebral drug infusions, when used, were targeted at the dorsal hippocampus.

## Results

### Everyday Memory.

**Fig. 1.**
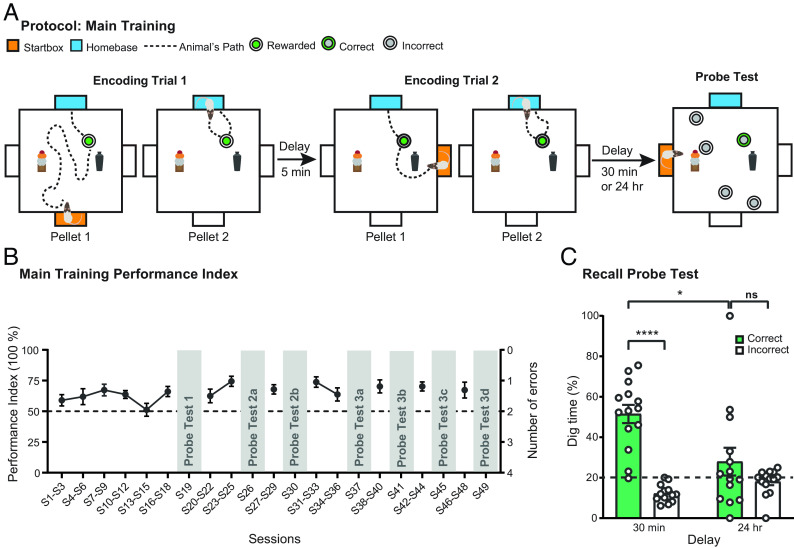
Everyday event arena protocol. (*A*) The training protocol consisted of 2 sample trials (STs) followed by a probe trial. During ST1, different startboxes (orange) were used for the first pellet and rats were trained to dig for pellets and carry these (dotted lines show representative trajectories) to a fixed home-base (blue). When retrieving the second pellet, rats left from the same home-base and returned with the pellet. ST2 was the same as ST1 but a different startbox was used. After a delay (either 30 min or 24 h), a CT1 was performed to determine whether the rats preferentially dug at the correct location. (*B*) Rats (*n* = 14) learned the task and maintained a stable above-chance performance from session 16 (S16). (*C*) Memory retention declined from well above chance at 30 min to chance level at 24 h (*n* = 14). Overnight forgetting is an important characteristic of episodic-like everyday memory. Means ± SEM and individual animal data plots. Dashed line (in *B* and *C*) indicates chance level. ns, not significant. *P < 0.05, ***P < 0.001.

The everyday memory task involves the encoding, storage, and forgetting of memory for a daily event in an arena. The concept of “everyday” memory is precisely of those things that happen during the day that we remember for a while but ordinarily forget; that is, both memory and forgetting are built into its definition. We achieved both memory and forgetting by training rats to search and dig for a small food reward in the sandwells located in different places each day. Fig. 1*A* shows a representative sequence of events in which, on sample trial 1 (ST1), an animal is placed in a startbox (orange) from which it enters the arena to find a single sandwell in a specific location. Once it has found the correct sandwell, dug through the sand and retrieved the 0.5 g food pellet, it then carries it to the home-base (blue) to eat. A second run within that trial then starts from this home-base with the animal returning to the sandwell and then back to the home-base with its second food pellet. ST2 then starts from a different starting location (orange, note change of startbox location) after minimal delay and the event sequence of “search-dig-retrieve-run” to the home-base is repeated, with a second run back to the sandwell from the home-base location also. After an interval of 30 min, a choice trial (CT) is scheduled in which, now, several sandwells are available of which only the sample location provides access to food. Performance is measured in terms of correct choices of this sandwell transformed into the performance index (PI). As the choice of sandwell location changes daily, the recency memory demand is “event-like” in character, particularly with the additional component of searching and digging.

Quantitative analysis showed that, over 49 sessions, it was possible to derive a PI with respect to memory on CTs (Fig. 1*B*). The PI rose significantly from an initial chance level (50%) to circa 75% by session 25 (S25), a level maintained through to S49 (ANOVA across S1-S29: F = 2.40, df 13/104, *P* < 0.01); this asymptotic level remained stable from S31 to S49 (F = 0.85, df 13/52, n.s.), a stability that was important as successive probe tests (PTs) were scheduled on selected sessions. PT2a and 2b (Fig. 1*C*) show the proportion of time spent digging at the correct location vs. incorrect locations after memory delays of 30 min or 24 h (time delays counterbalanced across the two PTs). Memory was highly significantly above chance at 30 min (51.48% ± 4.51, mean ± SEM) but not at 24 h (27.91% ± 6.87) (30-min group: one-sample *t* test vs. chance, t = 6.97, df = 1/8, *P* < 0.001; 24-h group: n.s.), with significant forgetting between these two retention intervals (F = 13.15, df 1/13, *P* < 0.01). The absolute dig time across multiple PTs (*SI Appendix*, Fig. S1) was circa 30–40 s during early PTs, and it showed a modest nonsignificant trend toward declining on later PTs; (F < 1, n.s.). What changed in different PTs described below was how the animals distributed their digging effort across the 5 sandwells—reflecting memory—rather than the total time digging. We therefore judged that the use of normalized data for the PTs was appropriate.

### Novelty Exposure Enhanced Memory Retention.

A key issue was whether novelty would enhance retention over time. In S92–S96, further STs were scheduled that were accompanied by 5-min novelty exploration (or a control condition) before or after the STs with memory tested in PT7 after 24 h (*SI Appendix*, Fig. S2 *A* and *B*). These gave the opportunity to see whether the retention of weakly encoded memory about where successful digging had happened recently could be boosted by novelty. As shown previously (14, 15), novelty exposure enhanced memory retention at 24 h relative to chance (average pre- and postconditions, F = 7.35, df 1/7, *P* < 0.05). In addition, we also performed a control test during recall in which intra- and extra-maze cues were occluded and found that performance declined to chance level (F < 1, n.s.) (*SI Appendix*, Fig. S2*C*). This test confirms the allocentric nature of this recency memory protocol.

### Viral Expression of Red-shifted Channelrhodopsin, Histological Confirmation, and Optogenetic Activation of LC-TH^+^ Neurons.

**Fig. 2.**
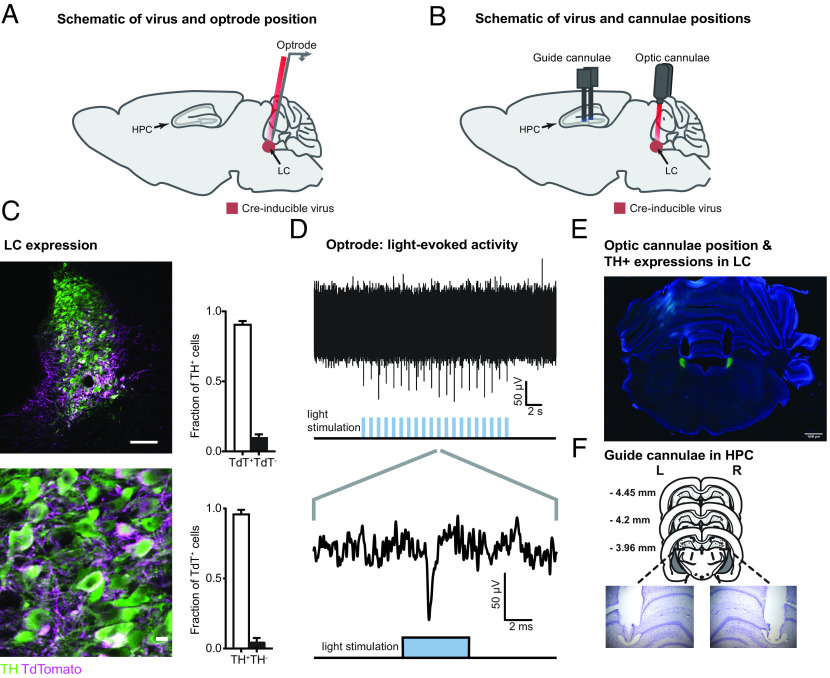
Histological confirmation and optogenetic activation of LC-TH^+^ neurons. (*A*) Schematic showing the placement of an optrode (optic fiber and electrode) used to record light-evoked multiunit firing in LC-TH^+^ neurons of Th-Cre rat expressing Cre-inducible AAV-DJ ChrimsonR-tdTomato (ChR^+^) virus. (*B*) Schematic showing the placement of bilateral optic cannulae in the LC and bilateral guide cannulae in the dorsal hippocampus (HPC) of Th-Cre rat. (*C*) Overlap between TH^+^ neurons and neurons expressing ChrimsonR-tdTomato at the position of the optic cannula in the LC. (Scale bars: *Upper*, 100 μm; *Lower*, 10 μm.) (*D*) Example of multiunit responses to optical stimulation recorded in the LC. The *Upper* panel shows multiunit spikes elicited by a train of light stimuli (20 5-ms pulses delivered at 1 Hz; note that each light pulse elicits a response). The *Lower* panel shows an expanded view of the multiunit response to a single pulse of light stimulation. (*E*) A representative example of the bilateral optic cannula positions in the LC used in behavioral experiments. (Scale bar: 1 mm.) (*F*) Histological verification of the bilateral guide cannulae tip locations in HPC used for drug infusions.

As the activity of LC-TH^+^ neurons has been linked to novelty perception (15), we then asked whether activation of these neurons could mimic the beneficial effect of novelty on memory retention. To achieve this, we injected a Cre-inducible adeno-associated virus (AAV) expressing the red-shifted channelrhodopsin ChrimsonR (hereafter ChR) (28) fused with tdTomato into the LC of Th-Cre rats (27). We then validated and used the light-sensitivity of ChR to elicit activation of LC-TH^+^ neurons (Fig. 2 *A* and *B*). We confirmed a high degree of overlap between TH^+^ neurons and neurons expressing ChR (ChR^+^) in the LC (Fig. 2*C*), with 89 ± 3% (mean ± SEM) of TH^+^ neurons expressing ChR, and 96 ± 1% of ChR^+^ neurons positive for the TH antigen (*n* = 4). We also confirmed that the axons originating from LC-ChR^+^ neurons reached the hippocampus, as indicated by positive staining for norepinephrine transporter (NET) and TH, confirming their LC origin as well as neuronal identity (*SI Appendix*, Figs. S3 and S4). By recording multiunit activity in the LC using an optrode, we demonstrated the ability to activate LC-ChR^+^ neurons with red light (Fig. 2 *A* and *D*). Light-evoked responses were recorded from three rats, and in all cases, multiunit activity was observed at a depth corresponding to the entire LC body (Fig. 2*D*). We then proceeded, in other animals, to implant bilateral optic cannulae to deliver light to the LC in order to determine the effect of light activation to LC-TH^+^ neurons in behavioral experiments (Fig. 2 *B* and *E*). Additionally, bilateral guide cannulae for drug infusions were inserted in the dorsal hippocampus to reach the CA1/DG border (Fig. 2*F*).

### Hippocampal Electrophysiology and Pharmacology.

**Fig. 3.**
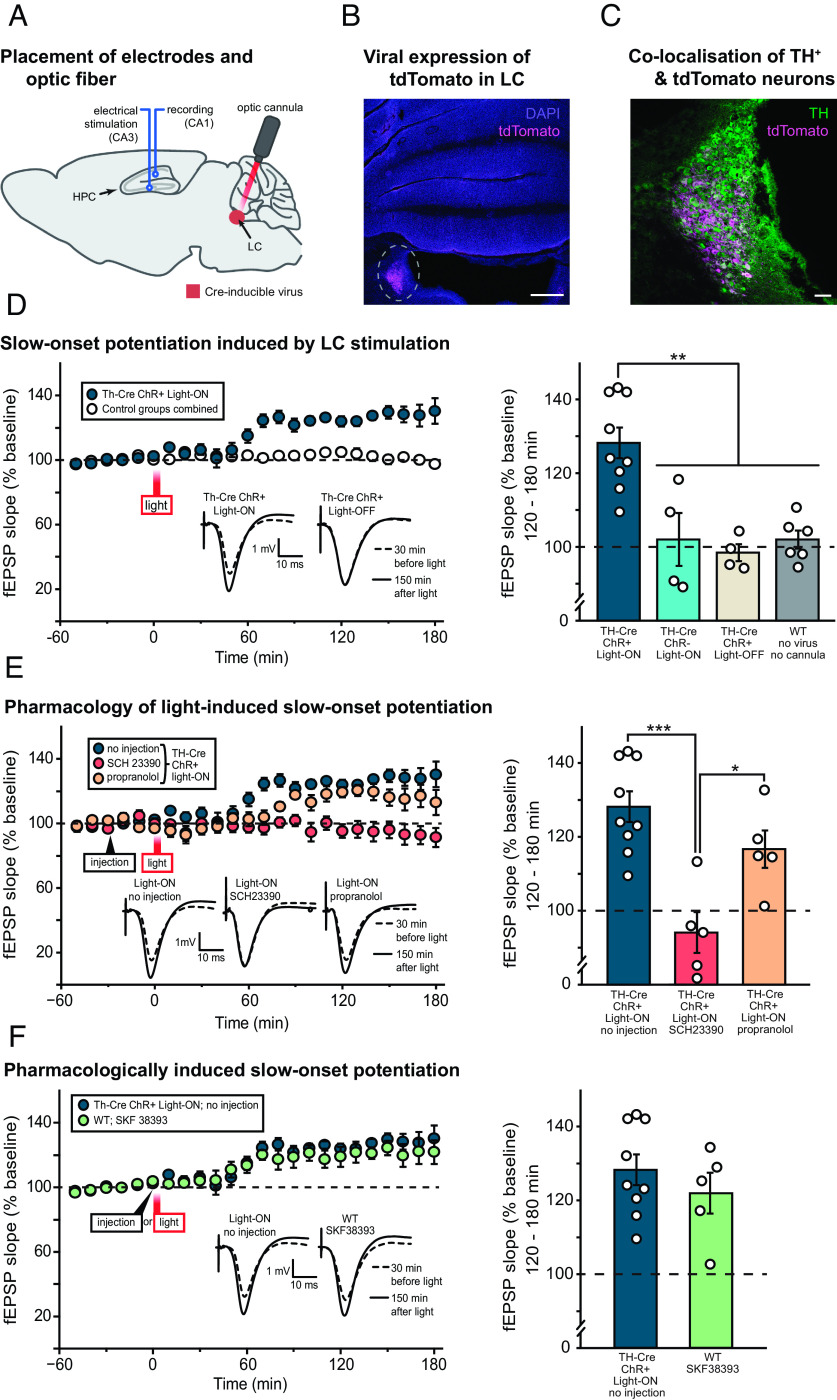
Optical stimulation of LC-TH^+^ neurons causes slow-onset potentiation (SOP) of CA1 fEPSPs in vivo. (*A*) Illustration of the placement of hippocampal stimulating and recording electrodes in CA3 and CA1 and an optic cannula in the LC of Th-Cre rat. (*B*) Representative example of tdTomato staining indicating viral expression in the LC beneath the location of the optic cannula, against a background of DAPI staining. The histology also showed that all optic fibers were correctly placed. (Scale bar: 500 μm.) (*C*) Higher magnification image of the same region, with TH and tdTomato staining overlaid. Similar colocalization of these two signals was observed in all the brains examined (a sample of 17 brains out of a total of 27, distributed across all groups). (Scale bar: 100 μm.) (*D*) The left-hand panel shows the time-course of SOP in the Th-Cre ChR^+^ Light-ON group (*n* = 9) plotted relative to the mean data from 14 rats comprising three control groups [Th-Cre ChR^–^ Light-ON (*n* = 4), Th-Cre ChR^+^ Light-OFF (*n* = 4), and WT with no virus injection or cannula implantation (*n* = 6)]. Mean fEPSP slope data were analyzed over 10-min time periods and normalized to the 1-h period before optical stimulation of the LC (light). Representative examples of fEPSPs recorded circa 30 min before and 150 min after light stimulation are shown. The right-hand panel shows mean fEPSP slope potentiation 120 to 180 min after light stimulation in the Th-Cre ChR^+^ Light-ON group and the three control groups, now plotted separately. **P < 0.01 in all cases, post hoc Tukey’s pairwise comparisons. (*E*) The left-hand panel shows the impact of systemic injection of SCH 23390 (1 mg/kg of body weight, *n* = 5) and propranolol (6.25 mg/kg of body weight, *n* = 5) on SOP induced by optical stimulation of LC-TH^+^ neurons in Th-Cre ChR^+^ rats analyzed and plotted as in panel *D*. The “no injection” control group (the same Th-Cre ChR^+^ Light-ON group from panel *D*) is reproduced for comparison. Representative examples of fEPSPs recorded circa 30 min before and 150 min after light stimulation are shown. The right-hand panel shows mean fEPSP slope potentiation 120 to 180 min after light stimulation in the no injection, SCH 23390, and propranolol groups. *P < 0.05, ***P < 0.001; post hoc Tukey’s pairwise comparisons. (*F*) The left-hand panel shows the time course of SOP induced by systemic injection of SKF 38393 (3 mg/kg of body weight, *n* = 5), relative to SOP observed in the no injection group (the same Th-Cre ChR^+^ Light-ON group from panels *D* and *E* reproduced for comparison). Representative examples of fEPSPs recorded circa 30 min before and 150 min after light stimulation are shown. The right-hand panel shows mean fEPSP slope potentiation 120 to 180 min after light stimulation in the no injection and SKF 38393 groups. Means ± SEM and individual animal data plots. Dashed line in panels *D*–*F* indicates baseline fEPSP slope.

The next step entailed optogenetic stimulation of LC-TH^+^ neurons of Th-Cre rats in parallel with evoked potential stimulation and recording in hippocampus in vivo, with an intracannula optic fiber targeting the LC and both stimulating and recording electrodes placed in CA3 and CA1, respectively (Fig. 3*A*). We again confirmed both the expression of ChR-tdTomato and its overlap with TH staining in the LC (Fig. 3 *B* and *C*).

A key finding was that activation of LC-TH^+^ neurons caused the induction of slow-onset potentiation (SOP) of field excitatory postsynaptic potentials (fEPSPs) in CA1 elicited by electrical stimulation of CA3 (Fig. 3 *D*–*F*). Fig. 3*D*, left-hand panel, shows the time-course of SOP after optical stimulation in the Th-Cre ChR^+^ Light-ON group plotted, for clarity, relative to the average of the three control groups [Th-Cre ChR^–^ Light-ON, Th-Cre ChR^+^ Light-OFF, and wild-type (WT) rats with no virus injection or cannula implantation]. Th-Cre ChR^–^ animals were infected with control viruses expressing only tdTomato in LC-TH^+^ neurons. SOP reached a peak in the second hour after light stimulation (60 trains of 20 pulses at 25 Hz; intertrain interval = 5 s) and was maintained for the 180-min duration of the experiment (c. 120–130% relative to the 100% baseline). Representative examples of fEPSPs recorded 30 min before and 150 min after light stimulation illustrate its impact on the slope and amplitude of the evoked response. Fig. 3*D*, right-hand panel, shows the mean normalized fEPSP slope over the final hour of recording, i.e. 120 to 180 min after stimulation, in the Th-Cre ChR^+^ Light-ON group versus the three control groups, this time plotted separately. There was a significant main effect of group (F = 12.39, df 3/19, *P* < 0.001), and post hoc pairwise comparisons (Tukey’s HSD) revealed the Th-Cre ChR^+^ Light-ON group to be significantly higher than each of the control groups (*P* < 0.01 in all cases). No group differences were found among the control groups (*P* > 0.9 in all cases). Potentiation in the Th-Cre ChR^+^ Light-ON group was also significantly above baseline exactly 120 to 180 min after stimulation (t_8_ = 6.79, *P* < 0.001; one-sample *t* test with Bonferroni correction for multiple comparisons), but no significant potentiation above baseline was observed in any of the control groups (ts < 1 in all cases).

The pharmacological sensitivity of LC photoactivation-induced SOP was then investigated. Fig. 3*E*, left-hand panel, shows the impact of intraperitoneal (IP) injections of the dopamine D1/D5 receptor antagonist SCH 23390 and the β-adrenoceptor antagonist propranolol on light-induced SOP. SCH 23390 caused a complete block of SOP in the hippocampus. In contrast, a delayed onset but only partial blockade was observed after propranolol injection, relative to the Th-Cre ChR^+^ Light-ON group that is reproduced here for comparison. Mean potentiation 120 to 180 min after stimulation is shown in the right-hand panel of Fig. 3*E*. There was a significant main effect of group (F = 12.66, df = 2/16, *P* = 0.001), and post hoc pairwise comparisons (Tukey’s HSD) revealed significantly enhanced SOP in the noninjected Th-Cre ChR^+^ Light-ON group relative to the SCH 23390-injected group (*P* < 0.001) and between propranolol and SCH 23390 groups (*P* = 0.025). However, the difference between the noninjected Th-Cre ChR^+^ Light-ON group and the propranolol group was not significant 120 to 180 min after stimulation (*P* = 0.27), although a transitory but significant difference was seen 60 to 120 min after stimulation (*SI Appendix*, Fig. S5). Comparisons with baseline (100%, one-sample *t* tests with Bonferroni correction for multiple comparisons) revealed no significant potentiation in the SCH 23390-treated group (t_4_ = 1.08, *P* = 0.68) but a nonsignificant trend toward potentiation in the propranolol group 120 to 180 min after light stimulation (t_4_ = 3.29, *P* = 0.06; Fig. 3 *E*, right panel). This potentiation relative to the baseline reached significance only in the earlier period of 60–120 min after light stimulation in this group but it was not sustained (*SI Appendix*, Fig. S5).

As a final step, we sought to compare LC-TH^+^ light stimulation-induced SOP with pharmacologically-induced SOP (Fig. 3*F*, left-hand panel). The time-course and magnitude of the increase in fEPSP were similar in the uninjected Th-Cre ChR^+^ Light-ON group, and in WT rats injected with the dopamine D1/D5 receptor agonist SKF 38393. Mean potentiation 120–180 min after stimulation/injection is shown in Fig. 3*F*, right-hand panel. There was no significant difference between the two groups (t_12_ = 0.91, *P* = 0.38), and potentiation was significantly above baseline in SKF 38393-injected rats (t_4_ = 3.94, *P* = 0.017; one-sample *t* test) as it had been in the Th-Cre ChR^+^ Light-ON group. There were no group differences in baseline stimulation intensity or fEPSP parameters corresponding to Fig. 3 *D*–*F* (*SI Appendix*, Fig. S6).

### Memory Retention.

**Fig. 4.**
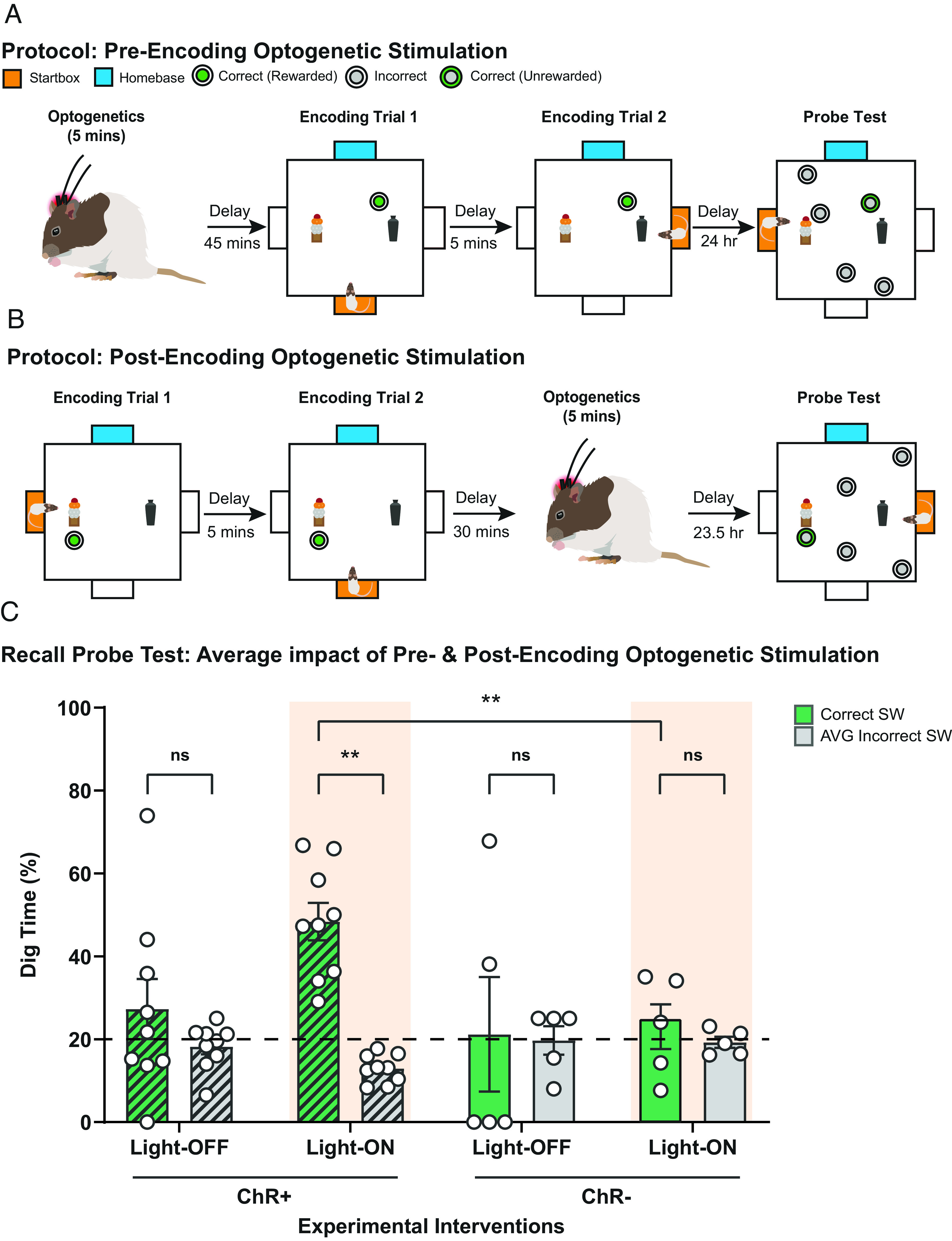
Optogenetic stimulation of TH^+^-LC neurons facilitates memory retention. (*A* and *B*) Experimental design for optogenetic preencoding (*A*) and postencoding (*B*) stimulation. Optogenetic stimulation of LC-TH^+^ neurons was scheduled either 45 min before memory encoding (*A*) in the event arena or, in a separate session, 30 min after encoding (*B*). A PT was scheduled 24 h later. (*C*) The Th-Cre ChR^+^ group that received optogenetic stimulation with red light (Light-ON) displayed good memory at 24 h (F = 7.48, df = 1/8, *P* < 0.01), and the Th-Cre ChR^+^ control group that did not receive optogenetic stimulation (Light-OFF) and in the Th-Cre ChR^–^ control animals were at chance level (1-sample *t* test, t = 6.26, df = 1/8, *P* < 0.001). Means ± SEM and individual animal data plots. Dashed line in panel *C* indicates chance level. ns, not significant. **P < 0.01.

Having established the electrophysiological consequences and pharmacological sensitivity of light stimulation of LC-TH^+^ neurons, we then turned our attention to the behavioral implications. Guided by the time-course of the observed SOP of fEPSPs in vivo, optogenetic stimulation of LC-TH^+^ neurons (60 trains of 20 pulses at 25 Hz; inter-train interval = 5 s) was scheduled either 45 min before memory encoding in the event arena or, in a separate session, 30 min after encoding (both being examined in all animals using a within-subjects, counterbalanced design). Fig. 4 *A* and *B* show the protocols for preencoding and postencoding stimulation of LC-TH^+^ neurons. In both cases, the PT for evaluating memory was not scheduled until 24 h later. Everyday memory of a changing reward location is usually forgotten within 24 h; this forgetting to baseline levels was observed in the Th-Cre ChR^+^ group that did not receive optogenetic stimulation (Light-OFF) and in the ChR^–^ control animals with Light-ON (Fig. 4*C*). In contrast, the Th-Cre ChR^+^ group receiving optogenetic stimulation with red light displayed good memory at 24 h, with all nine animals consistently showing above-chance memory (1-sample *t* test, t = 6.26, df = 1/8, *P* < 0.001; Fig. 4*C*). The Light-ON conditions also showed significantly better memory in the ChR^+^ group than the ChR^–^ group (F = 7.48, df = 1/8, *P* < 0.01).

### Pharmacological Interventions.

**Fig. 5.**
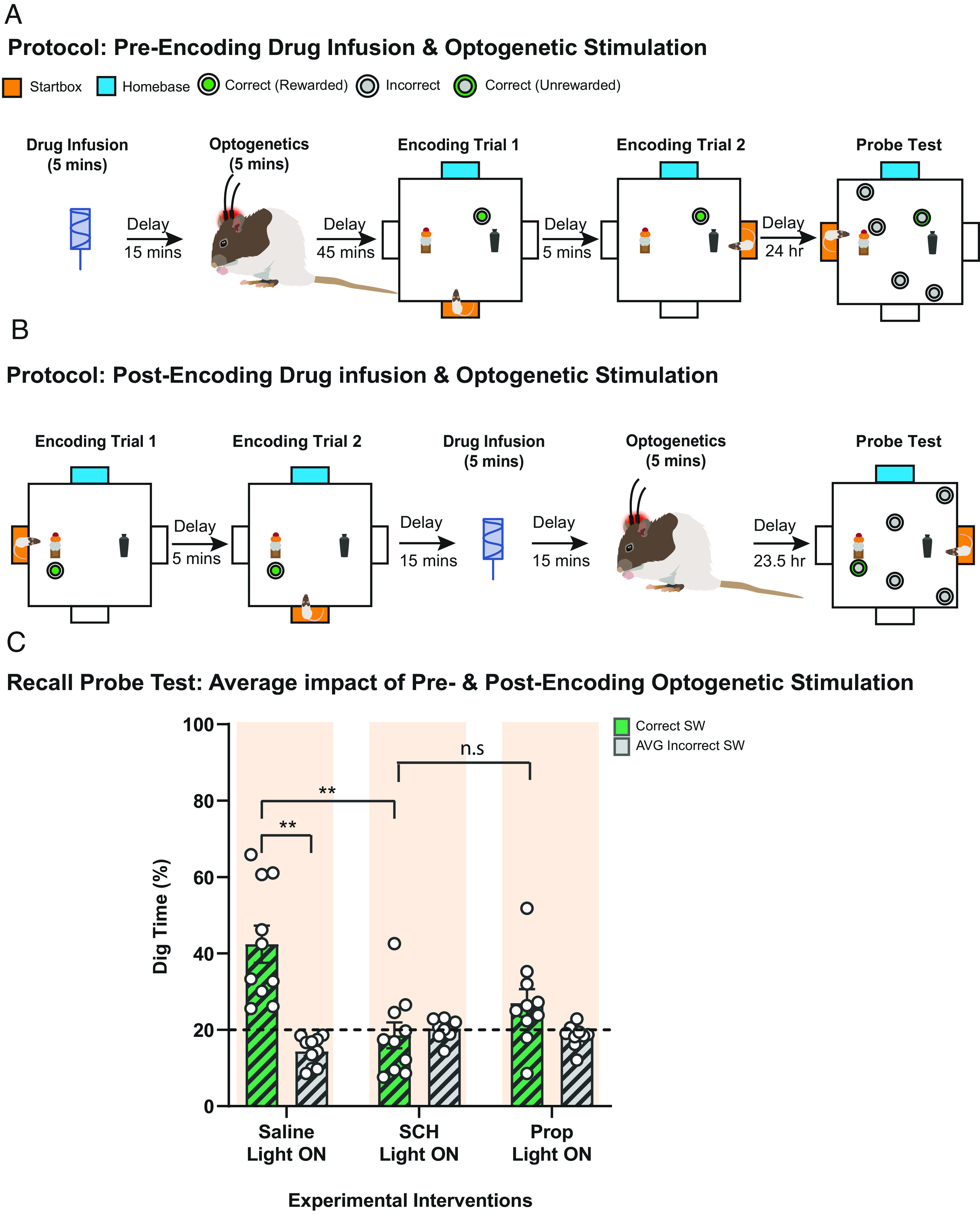
Pharmacological interventions in HPC and optogenetic stimulation of LC-TH^+^ neurons. (*A* and *B*) Experimental design for the pharmacological interventions and optogenetic preencoding (*A*) and postencoding (*B*) stimulation. Specific intrahippocampal drug infusions were administered 15 min prior to LC-TH^+^ stimulation, scheduled either before (*A*) or after (*B*) memory encoding during STs. (*C*) Blockade of hippocampal dopamine D1/D5 receptors (SCH) during LC-TH^+^ optogenetic stimulation abolished memory retention at 24-h PT. The effect of blockade of hippocampal β-adrenoceptor (Prop) was at an intermediate level, not significant than that for the control saline condition (n.s.). Means ± SEM and individual animal data plots. Dashed line in panel *C* indicates chance level. ns, not significant. **P < 0.01.

Although the LC is a noradrenergic brain region, previous findings have raised the possibility that, at least in the mouse, LC activation might paradoxically promote persistent memory by a dopaminergic rather than a noradrenergic mechanism (15, 29). To examine this matter further, we used protocols like those of Fig. 4, but with the addition of receptor-specific intrahippocampal drug infusions 15 min prior to LC-TH^+^ stimulation. These were scheduled either before or after event arena memory encoding during STs (Fig. 5 *A* and *B*). The impact of SCH 23390 and propranolol was investigated.

With light-activation of LC-TH^+^ neurons, excellent 24-h memory retention was seen after control intrahippocampal infusions of saline, comparable to that of Fig. 1, with all animals displaying above-chance memory (Fig. 5*C*). Memory retention was differentially affected by the three drug conditions (one-way ANOVA, F = 15.42, df = 1/9, *P* < 0.05). It was completely blocked by SCH 23390 (relative to chance: t = 0.43, df = 1/9, n.s.). In view of the time-course of the impact of these drugs on SOP of fEPSPs (Fig. 3), we also considered data subdivided with respect to preencoding vs. postencoding optogenetic activation. The positive trends apparent in Fig. 5*C* were replicated in each of these conditions (*SI Appendix*, Fig. S7), albeit with slightly greater individual subject variability. Finally, we double-checked that there would be no residual memory at 24 h in the saline condition with Light-OFF (*SI Appendix*, Fig. S7).

## Discussion

There are several key new findings of this study. First, rats readily learned the allocentric home-base version of the everyday memory task such that, at asymptote, they recalled the daily varying location at which they had most recently had an opportunity to dig up and eat a hidden food reward inside the arena. Performance fluctuated but was typically at 65 to 80% correct and consistently above chance. Second, in probe test data, both environmental novelty and optogenetic activation of histologically identified LC-TH^+^ neurons occurring either shortly before or after memory encoding enhanced the retention of memory to at least 24 h. Third, optogenetic activation of LC-TH^+^ neurons with a protocol mimicking phasic-activation of the LC caused SOP of concomitant glutamatergic fast synaptic transmission to a level of circa 15 to 25% above baseline, reaching an asymptote after c. 60 min. Fourth, both optogenetically enhanced memory retention and the SOP phenomenon were blocked by a dopamine D1/D5 receptor antagonist (SCH 23390), but only partially by a β-adrenoceptor antagonist (propranolol). These findings indicate that the retention of weak memory for recent events can be enhanced by perievent activation of catecholaminergic neuromodulatory neurotransmission in the rat, confirming and extending our earlier findings in the mouse.

Our overarching hypothesis is that forming a memory and retaining a memory have different determinants. Memory encoding is necessarily determined by events occurring at the precise moment an event happens, but whether new memory traces are retained or lost is determined by additional factors, one of which can be perievent neuromodulation that regulates the synthesis of PRPs. With respect to episodic-like memory, as in our spatial recency task, this neuromodulation might occur shortly before or after memory encoding. From a molecular perspective, memory encoding involves patterns of pre- and postsynaptic neural activity that induce N-methyl-D-aspartate (NMDA)-type glutamate receptor (NMDA receptor)-dependent potentiation in the hippocampus. This potentiation leads to a temporary structural enlargement of postsynaptic dendritic spines and the rapid insertion of additional α-amino-3-hydroxy-5-methyl-4-isoxazolepropionic acid (AMPA)-type glutamate receptors (AMPA receptors) at the postsynaptic density (PSD) via lateral movement along the lipid bilayer membrane of dendritic spines (8, 30). This structural and functional plasticity decays to baseline levels in the absence of PRPs and potentiation is lost (31). Perievent novelty associated with activation of LC-TH^+^ neurons in a phasic manner (16, 32), which we mimicked optogenetically, might activate a dopaminergic-dependent mechanism in the hippocampus that regulates translation of dendritically localized mRNAs which synthesize the PRPs over short segments of the nearby dendrite. The enlarged size of the dendritic spine may serve as a tag to capture these PRPs, which then help to stabilize both structural and functional changes according to the principles of STC.

Our first finding was of the encoding and then remembering, each day, of a new allocentrically defined location where food could be located, dug up, and carried to the home-base to be eaten. A memory of the daily changing location of a recent event was formed each session within the well-learned event-arena context that was stored in long-term memory. Recent memory traces normally decay to baseline within 24 h, providing the dual baseline of rapid memory encoding and daily forgetfulness with which to explore the impact of neuromodulation on retention. Various control conditions were deployed to check that behavioral performance was allocentric and uninfluenced by uncontrolled cues; these included probe trials in which no accessible food was present to check that spatial memory rather than any smell of available food at the correct sandwell was guiding memory recall (*SI Appendix*, Fig. S1*C*). The design of the sandwells and use of masking smells also ruled out mediation by olfactory artifacts, while a test involving removal of both intra- and extra-arena landmarks before a choice trial also caused performance to fall to chance establishing the veracity of allocentric coding (*SI Appendix*, Fig. S2*C*). The event arena is a powerful procedure in which memory recall during a choice or probe trial of the location of an earlier event is only of its most recent location and thus, most likely, hippocampus-based (23, 33). It might, for example, be mediated by an engram-related hippocampal index (34). Our earlier use of the event arena involved behavioral protocols that may have permitted egocentric encoding of recent information mediated by structures outside the hippocampus. The new protocol is dependent on hippocampal processing (23).

Second, our results confirm and extend our earlier findings with mice (15) in showing catecholaminergic modulation of memory retention following optogenetic activation of LC-TH^+^ neurons. They are consistent with Smith and Greene’s original findings (29), and observations in mice that dopamine release from terminals of LC-TH^+^ axons in the hippocampus is essential for both the updating of contextual recognition memory (35) and the linking of contextual fear memories formed at different times (36). Modulation given before as well as after memory encoding was examined. Modulation prior to memory encoding carries with it the possibility that there is a sensitizing effect on, for example, neuronal excitability that has been shown to affect allocation of memory traces (37). However, that similar enhancement of retention occurs with after-encoding modulation as well as the before-encoding condition mirrors electrophysiological observations made using both in vitro slice and in vivo studies of STC (7, 10). This is consistent with our proposed mechanism of enhanced retention being or at least including the capture of PRPs synthesized in the hippocampus in response to LC-TH^+^ neuron activation and their downstream effects on intracellular signal transduction pathways in the hippocampus.

Third, to our knowledge, the observation of SOP of fEPSPs in CA1 in vivo is the first demonstration following selective activation of LC-TH^+^ neurons, as first shown definitively both in vitro and in vivo in response to a dopamine D1/D5 receptor agonist (21, 38). SOP of fEPSPs and population spikes in vivo has been shown following brain-derived neurotrophic factor (BDNF) administration (39) and shown to require gene transcription (40). However, neurons whose activation can mimic such an effect in vivo had not previously been identified. The present in vivo phenomenon was also completely blocked by the application of the dopamine D1/D5 receptor antagonist SCH 23390. With respect to gain-of-function, we also confirmed in vivo that pharmacologically induced SOP of fEPSPs occurred after the administration of the dopamine D1/D5 receptor agonist SKF 38393 that was similar in magnitude and time-course to that observed after photostimulation of LC-TH^+^ neurons. These results are consistent with previous reports that dopamine D1/D5 receptor activation in vitro can cause a Ca^2+^-dependent potentiation of AMPA-type glutamate receptor-mediated evoked excitatory postsynaptic currents (EPSCs) (15, 41) and a slow-onset increase in evoked fEPSPs in the apical dendrites of CA1 in vitro; the latter phenomenon is dependent on protein synthesis, BDNF, and the activation of NMDA receptors, Ca^2+^/calmodulin (CaM)-dependent protein kinase II (CaMKII) and mitogen-activated protein kinase kinase (MEK) (21, 42–45). A complication, discussed elsewhere in detail (21, 43), is that the induction of lasting protein synthesis–dependent potentiation does not preclude the further induction of posttranslational early-LTP, an issue that has implications for behavioral significance (see below).

We also observed a slightly delayed onset and partial inhibition of LC-stimulation-induced SOP of fEPSPs after administration of the β-adrenoceptor antagonist propranolol. Potentiation was significantly reduced at 60–120 min, but not 120–180 min, after stimulation of LC-TH^+^ neurons. The apparently limited role of β-adrenoceptor is consistent with previous reports that exogenously or endogenously applied noradrenaline has little effect on EPSPs in CA1, despite causing an increase in neuronal excitability (46). Similarly, optogenetic activation of LC-TH^+^ neurons causes an SOP of population spike amplitude (but not fEPSP slope) in the dentate gyrus reminiscent of previous studies of noradrenaline-induced potentiation (47).

Our fourth finding centers on the pharmacology of our behavioral observations. Dopamine and noradrenaline share a common biosynthetic pathway, and corelease of both neurotransmitters occurs at the terminals of noradrenergic neurons of the LC (29). We here replicate in rats the key finding in mice of Takeuchi et al. ((15)) that dopamine D1/D5 receptors in the dorsal hippocampus, but not β-adrenoceptors, likely drive the facilitation of memory traces by distinct novel events which activate the LC (32). A different situation prevails in the amygdala where coactivation of glutamatergic and noradrenergic receptors is essential at the time of memory encoding (48). There are multiple ways in which the LC-stimulation-induced activation of CA1 dopamine D1/D5 receptors might facilitate the consolidation of memory, but distinct mechanisms make differing predictions about the effects of LC stimulation before or after encoding. Dopamine D1/D5 receptors couple via G-proteins to the cyclic adenosine monophosphate (cAMP)/cAMP-dependent protein kinase (PKA) signaling pathway, leading to increases in neuronal excitability, increases in synaptic NMDA receptor currents, the synthesis of PRPs, and stabilization of the synaptic insertion of AMPA receptors (49–51). The first option, increases in excitability and NMDA receptor currents, is arguably more consistent with a metaplastic facilitation of subsequent memory formation and memory allocation (19, 20). However, our observation that LC-TH^+^ stimulation is equally effective regardless of whether it is delivered before or after encoding favors a single parsimonious interpretation based on the STC framework. According to this hypothesis, dopamine release from catecholaminergic afferents provides a physiological trigger for the synthesis of PRPs before or after memory formation; these are then captured at synapses tagged at the time of memory encoding and the consequential rescue of decaying synaptic potentiation. In support of this view, pharmacological activation of dopamine D1/D5 receptors with SKF 38393 stimulates dendritic translation in cultured neurons in an NMDA receptor- and MEK-dependent manner (52, 53) and in vitro (21). That SOP does not occlude subsequent early LTP induction raises the possibility that, whether it occurs before or after memory encoding, EPSCs would be larger—a circumstance likely favoring more long-lasting memory.

An intriguing but speculative alternative is that dopamine D1/D5 receptors facilitate memory by increasing the synaptic expression of Ca^2+^-permeable AMPA receptors (CP-AMPARs) that lack the GluA2 subunit. Dopamine D1/D5 receptor activation causes PKA-dependent S845 phosphorylation of AMPA receptor GluA1 subunits and an increase in the cell-surface expression of GluA1-containing receptors (54, 55). Recent evidence suggests that activity-dependent posttetanus Ca^2+^ entry via CP-AMPARs is necessary for late-LTP (56). A complication is that heterosynaptic facilitation of potentiation of a weakly tetanized pathway was not observed when this activation preceded strong tetanization of a separate input (57). Nonetheless, in the latter study, a weak tetanus produced a small but nonetheless stable LTP regardless of the presence or absence of a subsequent strong tetanus. This result monitored over 3 h makes it difficult to dissociate the magnitude of LTP in the weak pathway from its persistence over time, a distinction that we have previously argued is central to the STC model in long time-scale experiments of 8 to 10 h (58).

Administration of the β-adrenoceptor antagonist propranolol also caused a modest impairment in the ability of LC-TH^+^ stimulation to facilitate memory retention, but the effect did not reach significance unless data from pre- and postencoding infusions were combined. We therefore cannot rule out a contribution of noradrenaline release from LC terminals in the hippocampus to the enhancement of recency memory in rats, in contrast to the lack of effect observed in our previous mouse study (15). However, a modest impairment such as this is consistent with the partial block of LC-TH^+^-light stimulation-induced SOP of fEPSPs by propranolol discussed above and the ability of β-adrenoceptor stimulation to facilitate the induction of late-LTP (59). We and others have previously argued that LC signals the occurrence of novel experiences that bear only a minimal relationship to past experiences (distinct novelty), whereas novel experiences that share some commonality with past ones (common novelty) are signaled by the ventral tegmental area (VTA) (32).

In the absence of dose-response data, we cannot entirely rule out the possibility that we have underestimated the role of β-adrenoceptors in our behavioral and electrophysiological experiments. However, the drug doses administered are comparable to those used in previous studies of memory and LTP. For the behavioral study, the doses of propranolol (6.25 µg per side) and SCH 23390 (1 µg per side) infused intrahippocampally were chosen to match those used by Takeuchi et al. ((15)). Our intracerebral propranolol dose is substantially higher than that reported to block the induction of dentate LTP in vivo (60) or to modulate memory consolidation (61). For the electrophysiological experiment, we used systemic IP administration, maintaining the same dose ratio of propranolol to SCH 23390 (propranolol: 6.25 mg/kg of body weight, SCH 23390: 1 mg/kg). Comparable systemic doses of propranolol have been shown to effectively impair novelty seeking and memory (62, 63). Additionally, a systemic dose of 5 mg/kg of SCH 23390 limits the persistence of CA1 LTP in anesthetized rats (64), while behavioral testing is not feasible at comparable systemic doses of SCH 23390 owing to the striatal actions of dopamine D1/D5 receptor antagonists.

A remaining puzzle is what causal role, if any, the SOP of fEPSPs induced by LC-TH^+^ stimulation plays in memory formation and retention. Our perspective is that a key aspect of dopamine D1/D5 receptor activation is that it triggers the synthesis of PRPs as shown by the rescue of subsequent weakly induced early-LTP into lasting late-LTP (43, 65). This critical finding is in line with our STC perspective on both the SOP phenomenon itself and the enhanced retention of memory. However, we recognize three qualifications. First, the mechanism of tag-setting is unclear in relation to SOP. One possibility is that NMDA receptor activity during test stimulation responsible for a background level of tag-setting recruits PRPs synthesized in response to dopamine D1/D5 receptor activation. Although the NMDA receptor is not typically thought to be activated by low-frequency stimulation, a small NMDA-receptor-dependent component of the fEPSP is sometimes observed, particularly in vivo (33). This possibility is consistent with the observation that SOP in vitro is an activity-dependent process that does not occur if baseline stimulation is paused after administration of SKF 38393 (21). However, this leads to a second qualification—we cannot be certain that SOP of CA3–CA1 synaptic strength occurs under natural circumstances in behaving rodents in the absence of artificial low-frequency test stimulation. Future electrophysiological and behavioral experiments in which electrical stimulation is paused after activation of LC-TH^+^ neurons, or ongoing synaptic activity is blocked by, for example, intrahippocampal lidocaine administration in vivo, would shed light on this aspect (21). Even if SOP does not occur under natural circumstances, it might still provide an index of the capacity for dopamine D1/D5 receptor activation to stabilize potentiation at tagged synapses.

The third qualification, assuming SOP of fEPSPs does occur in the awake animal, relates to the temporal order of SOP and memory encoding. LC-TH^+^ stimulation given prior to memory encoding should cause larger EPSCs at the moment of distributed memory encoding that could contribute to enhanced retention within such a network, as shown in human event-related functional MRI (fMRI) studies (1, 2). However, preencoding SOP may also contribute to the enhanced neural excitability that favors effective allocation of neurons to a memory engram (20, 36). These increases in excitability typically last 5 to 6 h after a learning event (20, 66, 67). It would be interesting to determine how closely this time window matches the duration of SOP of fEPSPs induced by dopamine D1/D5 receptor activation. Although for technical reasons, our recordings lasted only 3 h after LC-TH^+^ stimulation or SKF 38393 injection, the population mean showed no sign of decline at this point, and SOP induced by SKF 38393 in vitro has been recorded for 6 h (21). The situation is different when SOP is induced after memory encoding. Enhanced neural activity would then occur 30 to 45 min after memory encoding—i.e., when the animals would be back in their home cages and away from the event arena, which may limit the effectiveness of preferential allocation. The STC hypothesis, in contrast, has temporal symmetry with respect to the order of encoding (tag-setting) and novelty (PRP synthesis), with the only difference being the potential time-course of decay for synaptic tags to return to baseline and the dissipation of PRP availability. The overarching general issue here is that strength and persistence do not necessarily covary, a matter that is usually finessed in studies of LTP lasting only 1 to 2 h.

A radically different prediction might be that SOP should impair memory. SKF 38393-induced SOP in vitro occludes late-LTP induced by tetanic stimulation (42), suggesting that the two engage overlapping mechanisms. It would be potentially valuable to conduct analogous occlusion experiments using optogenetic LC-TH^+^ stimulation in vivo. However, there are practical difficulties to doing this arising from the sheer length of time such an experiment would take. Monitoring of STC process and late-LTP in vivo (10) requires a minimum of 3 h after LTP induction. This would then have to be followed by attempted induction of SOP which would also have to be monitored for 3 h. Animal preparation and baseline recording may then take the length of an experiment to >9–10 h. This is impractical unless done in freely moving animals which is not without additional difficulties. However, in view of the extensive measures required to achieve a sufficient level of electrical LTP induction to block memory formation or retention (68, 69), it is arguably unlikely that the SOP observed here would be expected to block or reverse memory. Indeed, drugs that cause a pharmacological SOP typically enhance hippocampus-dependent memory—including the dopamine D1/D5 receptor agonist studied here (70).

In conclusion, our observations contribute to a growing body of data that has explored the impact of cell firing in the LC on memory formation and retention (71), now thought to be mediated by distinct patterns of tonic and phasic activation (16). Our findings support the notion that neuromodulatory influences on dendritic mRNA translation and the provision of PRPs likely contribute causally to memory retention.

## Materials and Methods

The methods focus on the behavioral studies. Issues concerning viral vectors, in vivo optrode recordings, in vivo electrophysiology with optogenetic stimulation, drug administration, histology, and statistical analysis are in Supplementary Information.

### Blinding.

All critical tests were conducted “blind.” The procedures were overseen by the University of Edinburgh Ethical Review Committee and compliant with the UK Rats Scientific Procedures Act (1986) and European Communities Council Directive of 24th November 1986 (86/609/EEC) legislation, which governs the maintenance of laboratory rats and their use in scientific experiments.

### Subjects.

We used Long-Evans Th-Cre transgenic males (27) backcrossed 10 times to the Lister Hooded strain (Charles River) in the behavior and optogenetics experiments. The first and second cohort (Exp 1: *n* = 14, Exp 2: *n* = 10) were at least 4 mo old and weighed approximately 300 g at the start of experiments. In the electrophysiology and optogenetics studies, 30 Th-Cre^+/–^ rats and 11 Th-Cre^–/–^ littermates (250 to 600 g) were used. They were maintained on a 12-h light/12-h dark cycle, with all testing during the light phase of the cycle (7 am–7 pm). The rats had ad libitum access to water and were maintained at 85 to 90% of their free feeding body weight according to a laboratory-derived growth curve.

### Surgical Procedures.

Standard surgical procedures were used under isoflurane anesthesia. The animals were placed in a stereotactic frame (Kopf Instruments) using a flat skull position. Breathing rate, heart rate, and blood oxygenation level were monitored using the MouseOx Plus system (STARR Life Sciences) coupled to a foot sensor. There was a recovery period of 14 d before training began.

Virus was infused bilaterally (1 μL) into the LC coordinates from lambda: anterior–posterior (AP), –3.1 mm; mediolateral (ML), ± 1.2 mm; and dorsal–ventral (DV) from the dura at 2 depth, –5.80 mm and –6.00 mm (72) using a microsyringe (SGE) and pump (sp200i microsyringe pump, WPI), with 500 nL per site. After each injection, the needle was kept in place for 10 min to ensure virus diffusion.

In animals undergoing behavior experiments, two-ferrule optic bi-cannulae (2.4 mm pitch, 9 mm length; Doric lenses) were implanted immediately above the LC (coordinates from lambda: AP, –3.1 mm; ML, ± 1.2 mm; DV from the dura, –5.5 mm) at a 20° angle to the coronal plane. Guide cannulae for later drug infusions (26 gauge, 46 mm outer diameter; Plastics One) were implanted bilaterally into the dorsal hippocampus (from bregma: AP, –4.5 mm; ML, ± 3.0 mm; DV from the dura, –2.5 mm). These were fixed in place using dental cement and the headcap was secured to the skull using jeweler’s screws. To prevent blockages/infection, dummy stylets were inserted into the guide cannulas. After the surgery, Rimadyl (carprofen, 0.08 mL/kg) was administered by subcutaneous injection. All rats had a recovery period of at least 7 d for them to regain their presurgery weights before electrophysiological or behavioral testing.

### Behavioral Apparatus.

The event arena has been described previously (22, 23). It consisted of a square open field [160 cm (l) × 160 cm (w)] surrounded by four transparent plexiglass walls and two intraarena cues; several extraarena cues were positioned around its perimeter. There were five locations within the arena where a sandwell could be inserted. Before and after each trial of every session, the event arena floor was cleaned with a 70% ethanol solution to prevent cryptic olfactory cues. Luminosity was maintained within 115 to 125 lx and temperature between 21 and 24 °C. There were four black entry/goal boxes with remotely controlled doors around the arena from which or to which the animals could run (Fig. 1). The black box on the “North” wall was used as an allocentrically stable “home-base” and the other 3 black boxes were used as “startboxes.” These boxes allowed the rats access to the arena at the start of each trial but they had to run to the home-base with food dug from the sandwells. The sandwells had an accessible and a deeper inaccessible section, separated by a curved, perforated plastic sheet. All sandwells within the event arena (one rewarded and four unrewarded) contained the same number of food pellets (12 pellets, 0.5 g/pellet; BioServ). The rewarded sandwell contained 4 food-reward pellets in the accessible section and 8 pellets in the inaccessible section; unrewarded sandwells had all 12 pellets in the inaccessible section. As a further masking odor, garam masala was added to the sand used to fill the sandwells (40 g garam masala per 4 kg sand). The ratio of garam masala to sand was measured and renewed at the start of each session, ensuring stable masking.

### Experimental Controls and Counterbalancing.

Rats were allocated randomly to the experimental or a control virus group. Several counterbalancing measures (e.g., sandwell locations across sessions) were implemented in this experiment (22, 23). Together, these measures prevented the same rewarded location and sandwell set from being trained successively. Furthermore, to avoid bias when observing the effect of the experimental interventions, the trials were recorded by an experimenter who was blind to the intervention applied to each animal.

### Everyday Memory Protocol.

The key features are “sample” trials (ST) each day with only one sandwell, delineating the correct location for accessible food in that session; followed by a “choice” trial (CT) or probe trial (PT) during which, in choosing between five sandwells, the animals display some level for memory for where the food was positioned most recently. As no local recognition cues identify the correct sandwell location, a choice test requires memory recall for a recent event (episodic-like memory). A second key feature is that once the strategy of the task is learned, successive sessions can run semiindefinitely over weeks at typically a stable performance level of around 75% correct. A total of 150 sessions using this protocol were performed with numerous PTs interleaved within this asymptotic level (cohort 1: S1–S50; cohort 2: S1–S100). The daily “event” consists of finding and digging up the food from different places each day during a ST and then carrying it to the safe home-base to eat, with memory of the changing locations where this happens across days being tested at various time-delays later using CTs or PTs (i.e., recency memory).

It is important to emphasize that the animals learn anew each day. While many tasks have a learning phase extending over several days and then a separate later recall phase, this “everyday” recency memory protocol has both during each daily session.

### Habituation.

Cohort 1 (*n* = 14) and cohort 2 (*n* = 10) were each habituated to the event arena for a total of 7 sessions. In the first habituation session (H1), rats were given 10 min to explore the event arena and the intraarena cues. In H2, one sandwell containing one accessible pellet (0.5 g) was placed at a random location within the arena. The rats were first “cued” in a startbox with one pellet and then, after 30 s, the startbox door was opened, allowing the rats to enter the arena and retrieve the pellet placed on top of the sandwell. Once this pellet was recovered, the rats were required to locate and enter the North home-base to eat the pellet. In the following sessions (H3–H7), the number of pellets and the depth at which they were placed within a sandwell were increased to encourage digging. By the seventh session (H7), all four pellets were placed at the bottom of the accessible section; this was the rewarded sandwell setup used throughout main training with its location altered across H2–H7.

### Main Training.

Every main training session consisted of two STs (ST1, ST2) followed, after a delay of approximately 30 min, by a recall CT1. During each ST, rats were trained to locate and retrieve two of the four accessible pellets (0.5 g per pellet) from the correct sandwell and then enter the North home-base to eat each one.

At the start of each trial, rats were placed in one of the allocated startboxes (e.g., ST1: East, ST2: South) where they were given one “cue” pellet (0.5 g/pellet). Once in the arena, rats were trained to find the correct sandwell and retrieve the 1st pellet. After eating it in the North home-base, they reentered the arena to retrieve a 2nd pellet from the same correct sandwell. After a 30-min delay, there was a CT1 where rats were placed in the remaining unused startbox of the session (e.g., CT1: West) and rewarded for finding the correct sandwell amongst the then five sandwells. The latency (s) to retrieve the correct pellet, the duration of digging (s), and the sequential order of visited sandwells (choice) were meticulously recorded. We also recorded errors, defined as the number of incorrect sandwells visited before locating the correct sandwell during CTs. A “performance index (PI)” was calculated using: PI (%) = [(maximum number of errors – actual number of errors)/(maximum number of errors)] × 100. This measure is at 50% for chance performance, with higher scores indicating good performance.

### Recall Probe Tests.

Periodically throughout the experiment, recall PTs with no accessible food were scheduled to examine memory retention for the correct sandwell location following various experimental interventions [cohort 1: S19 (PT1), S26 (PT2a), S30 (PT2b), S37, S41, S45, S49 (PT3a-d); cohort 2: S25 (PT1), S32 (PT2a), S36 (PT2b), S43, S47, S51 (PT3a-c), S55, S59, S63, S67 (PT4a-d), S71 (PT5), S75 (PT6)]. Three pellets were placed in the correct Sandwell at the end of the test to avoid extinction. These PTs were only performed if the average PI for three consecutive sessions of main training was at or above 60% (typically it was much higher). Recall PTs began with two STs followed by a recall PT after a delay of either 30 min or 24 h. None of the five sandwells were rewarded during this test, and the rats allowed 120 s to search amongst them. During this time, latency (s) to retrieve the correct pellet, dig times at each sandwell and order of sandwells visited were recorded. Both the absolute and normalized proportion of time at the correct and incorrect sandwells were calculated.

### Spatial Strategy Probe Test.

A spatial strategy PT was performed [Cohort 2: S84 (PT7a), S88 (PT7b)] to test whether use of the stable home-base successfully encouraged rats to employ an allocentric spatial strategy. During the STs, intra- and extra-arena spatial cues were present. After a short delay (~30 min), rats performed the PT in which the intra- and extra-arena spatial cues were removed and the arena rotated by 45° anti-clockwise and surrounded by a white curtain. Performance based on an allocentric strategy should fall to chance under these conditions.

### Optogenetic Stimulation.

A primary aim of the study was to explore the impact of optogenetic stimulation of LC-TH^+^ neurons upon memory retention which was administered either 45 min before or 30 min after encoding in a within-subject design. Rats were habituated to the optogenetic procedure (5 × 10-min sessions) to minimize the intrinsic novelty and stress of the procedure itself (which we judged to be minimal). During stimulation, rats were placed in a transparent plexiglass bowl [40 cm (d) × 37 cm (h)], which provided sufficient room to move naturally (twenty 5-ms pulses of 590-nm light at 25 Hz, delivered every 5 s for a total of 5 min). This was performed using two Fiber-Coupled LEDs (10 mW) connected to two single-fiber optic patch cords (2.5 m length, Thorlabs) and an optic driver (LEDD1B, Thorlabs). Both LEDs were synchronously controlled using custom LabView software.

### Drug Infusions Associated with Optogenetic Interventions.

Drug infusions were performed 15 min before optogenetic stimulation. Rats were also habituated to the drug infusion procedure for two sessions. A microinfusion pump (sp200i) was used for bilateral infusion (1 μL per hemisphere; 0.25 μL/min flow rate) of either dopamine D1/D5 receptor antagonist SCH 23390 (SCH), β-adrenoceptor antagonist propranolol (Prop), or sterile saline (Vehicle). SCH 23390 hydrochloride (324.24 g/mol, Tocris Bioscience) and (S)-(−)-propranolol hydrochloride (295.80 g/mol, Sigma-Aldrich) were dissolved in sterile saline (Sigma-Aldrich) and kept in frozen aliquots (50 μL) until use. Final drug concentrations were 3.1 mM (1 μg/μL) for SCH 23390 and 21.1 mM (6.25 μg/μL) for propranolol. Microsyringes (5 μL, SGE) were mounted on the microinfusion pump and connected via flexible polyethylene tubing to the injection cannulas (33-gauge, 0.5 mm beyond the implanted guide cannulas). During infusions, rats were gently restrained using a towel. The injection cannulas were left in the guide cannulas for 2 min after each infusion ended and the dummy cannulas replaced.

### Novelty Exploration.

The object of the optogenetic stimulation is to mimic patterns of neural activity expected in the LC in association with perievent novelty (e.g., unexpected experience). It was therefore advisable to determine separately the effect of novelty itself. This was done for both the preencoding and postencoding conditions. The rats were placed, for the first time, in a square transparent plexiglass box [100 cm (l) × 100 cm (w) × 30 cm (h)] lined with a novel floor substrate (pinecones) in the center of the event arena 45 min before the daily STs. To explore the effect of novelty (postencoding), rats were similarly placed in a star-shaped transparent plexiglass box [110 cm (l) × 110 cm (w) × 30 cm (h)], lined with a novel floor substrate (balloons) 30 min after the STs. A recall PT was performed after a delay of 24 h, with the prediction that memory for the recently correct place would be remembered well.

## Supplementary Material

Appendix 01 (PDF)

Dataset S01 (XLSX)

## Data Availability

All data and additional methods are included in the article and/or supporting information, Dataset S1.

## References

[1] A. D. Wagner , Building memories: Remembering and forgetting of verbal experiences as predicted by brain activity. Science **281**, 1188–1191 (1998). 9712582 10.1126/science.281.5380.1188

[2] J. B. Brewer, Z. Zhao, J. E. Desmond, G. H. Glover, J. D. Gabrieli, Making memories: Brain activity that predicts how well visual experience will be remembered. Science **281**, 1185–1187 (1998). 9712581 10.1126/science.281.5380.1185

[3] R. Brown, J. Kulik, Flashbulb memories. Cognition **5**, 73–99 (1977).

[4] W. Hirst , Long-term memory for the terrorist attack of September 11: Flashbulb memories, event memories, and the factors that influence their retention. J. Exp. Psychol. Gen. **138**, 161–176 (2009). 19397377 10.1037/a0015527 PMC2925254

[5] F. Ballarini, M. C. Martinez, M. Diaz Perez, D. Moncada, H. Viola, Memory in elementary school children is improved by an unrelated novel experience. PLoS One **8**, e66875 (2013). 23840541 10.1371/journal.pone.0066875 PMC3686730

[6] U. Frey, R. G. M. Morris, Synaptic tagging and long-term potentiation. Nature **385**, 533–536 (1997). 9020359 10.1038/385533a0

[7] U. Frey, R. G. M. Morris, Synaptic tagging: Implications for late maintenance of hippocampal long- term potentiation. Trends Neurosci. **21**, 181–188 (1998). 9610879 10.1016/s0166-2236(97)01189-2

[8] R. L. Redondo, R. G. M. Morris, Making memories last: The synaptic tagging and capture hypothesis. Nat. Rev. Neurosci. **12**, 17–30 (2011). 21170072 10.1038/nrn2963

[9] A. Govindarajan, I. Israely, S. Y. Huang, S. Tonegawa, The dendritic branch is the preferred integrative unit for protein synthesis-dependent LTP. Neuron **69**, 132–146 (2011). 21220104 10.1016/j.neuron.2010.12.008 PMC3032443

[10] K. L. Shires, B. M. Da Silva, J. P. Hawthorne, R. G. Morris, S. J. Martin, Synaptic tagging and capture in the living rat. Nat. Commun. **3**, 1246 (2012). 23212375 10.1038/ncomms2250 PMC3705498

[11] M. Z. B. Ibrahim, A. Benoy, S. Sajikumar, Long-term plasticity in the hippocampus: Maintaining within and “tagging” between synapses. FEBS J. **289**, 2176–2201 (2022). 34109726 10.1111/febs.16065

[12] J. Pinho, C. Marcut, R. Fonseca, Actin remodeling, the synaptic tag and the maintenance of synaptic plasticity. IUBMB Life **72**, 577–589 (2020). 32078241 10.1002/iub.2261

[13] D. Moncada, H. Viola, Induction of long-term memory by exposure to novelty requires protein synthesis: Evidence for a behavioral tagging. J. Neurosci. **27**, 7476–7481 (2007). 17626208 10.1523/JNEUROSCI.1083-07.2007 PMC6672624

[14] S. H. Wang, R. L. Redondo, R. G. Morris, Relevance of synaptic tagging and capture to the persistence of long-term potentiation and everyday spatial memory. Proc. Natl. Acad. Sci. U.S.A. **107**, 19537–19542 (2010). 20962282 10.1073/pnas.1008638107 PMC2984182

[15] T. Takeuchi , Locus coeruleus and dopaminergic consolidation of everyday memory. Nature **537**, 262–357 (2016). 10.1038/nature19325 PMC5161591 27602521

[16] C. W. Harley, Q. Yuan, Locus coeruleus optogenetic modulation: Lessons learned from temporal patterns. Brain Sci. **11**, 1624 (2021). 34942924 10.3390/brainsci11121624 PMC8699422

[17] N. K. Totah, R. M. Neves, S. Panzeri, N. K. Logothetis, O. Eschenko, The locus coeruleus is a complex and differentiated neuromodulatory system. Neuron **99**, 1055–1068.e6 (2018). 30122373 10.1016/j.neuron.2018.07.037

[18] N. K. Logothetis , Hippocampal-cortical interaction during periods of subcortical silence. Nature **491**, 547–553 (2012). 23172213 10.1038/nature11618

[19] D. J. Cai , A shared neural ensemble links distinct contextual memories encoded close in time. Nature **534**, 115–118 (2016). 27251287 10.1038/nature17955 PMC5063500

[20] S. A. Josselyn, P. W. Frankland, Memory allocation: Mechanisms and function. Annu. Rev. Neurosci. **41**, 389–413 (2018). 29709212 10.1146/annurev-neuro-080317-061956 PMC9623596

[21] S. Navakkode, S. Sajikumar, J. U. Frey, Synergistic requirements for the induction of dopaminergic D1/D5-receptor-mediated LTP in hippocampal slices of rat CA1 in vitro. Neuropharmacology **52**, 1547–1554 (2007). 17433377 10.1016/j.neuropharm.2007.02.010

[22] N. Broadbent , A stable home-base promotes allocentric memory representations of episodic-like everyday spatial memory. Eur. J. Neurosci. **51**, 1539–1558 (2020). 31944427 10.1111/ejn.14681 PMC7614820

[23] D. Tse, A. C. Norton, P. A. Spooner, R. G. M. Morris, A behavioral task modeling “everyday memory” in an event arena to foster allocentric representations for rodents. J. Vis. Exp., 10.3791/63152 (2022). 35188115

[24] S. S. Deshmukh, J. J. Knierim, Influence of local objects on hippocampal representations: Landmark vectors and memory. Hippocampus **23**, 253–267 (2013). 23447419 10.1002/hipo.22101 PMC3869706

[25] C. Wang , Egocentric coding of external items in the lateral entorhinal cortex. Science **362**, 945–949 (2018). 30467169 10.1126/science.aau4940 PMC6261310

[26] C. Wang, X. Chen, J. J. Knierim, Egocentric and allocentric representations of space in the rodent brain. Curr. Opin. Neurobiol. **60**, 12–20 (2020). 31794917 10.1016/j.conb.2019.11.005 PMC7080648

[27] I. B. Witten , Recombinase-driver rat lines: Tools, techniques, and optogenetic application to dopamine-mediated reinforcement. Neuron **72**, 721–733 (2011). 22153370 10.1016/j.neuron.2011.10.028 PMC3282061

[28] N. C. Klapoetke , Independent optical excitation of distinct neural populations. Nat. Methods **11**, 338–346 (2014). 24509633 10.1038/nmeth.2836 PMC3943671

[29] C. C. Smith, R. W. Greene, CNS dopamine transmission mediated by noradrenergic innervation. J. Neurosci. **32**, 6072–6080 (2012). 22553014 10.1523/JNEUROSCI.6486-11.2012 PMC3371362

[30] D. Choquet, P. Opazo, The role of AMPAR lateral diffusion in memory. Semin. Cell Dev. Biol. **125**, 76–83 (2022). 35123863 10.1016/j.semcdb.2022.01.009

[31] Y. Yang, J. J. Liu, Structural LTP: Signal transduction, actin cytoskeleton reorganization, and membrane remodeling of dendritic spines. Curr. Opin. Neurobiol. **74**, 102534 (2022). 35398661 10.1016/j.conb.2022.102534

[32] A. J. Duszkiewicz, C. G. McNamara, T. Takeuchi, L. Genzel, Novelty and dopaminergic modulation of memory persistence: A tale of two systems. Trends Neurosci. **42**, 102–114 (2019). 30455050 10.1016/j.tins.2018.10.002 PMC6352318

[33] T. Bast, B. M. da Silva, R. G. M. Morris, Distinct contributions of hippocampal NMDA and AMPA receptors to encoding and retrieval of one-trial place memory. J. Neurosci. **25**, 5845–5856 (2005). 15976073 10.1523/JNEUROSCI.0698-05.2005 PMC6724786

[34] T. D. Goode, K. Z. Tanaka, A. Sahay, T. J. McHugh, An integrated index: Engrams, place cells, and hippocampal memory. Neuron **107**, 805–820 (2020). 32763146 10.1016/j.neuron.2020.07.011 PMC7486247

[35] D. K. Galvez-Marquez , Spatial contextual recognition memory updating is modulated by dopamine release in the dorsal hippocampus from the locus coeruleus. Proc. Natl. Acad. Sci. U.S.A. **119**, e2208254119 (2022). 36442129 10.1073/pnas.2208254119 PMC9894183

[36] A. Chowdhury , A locus coeruleus-dorsal CA1 dopaminergic circuit modulates memory linking. Neuron **110**, 3374–3388.e8 (2022). 36041433 10.1016/j.neuron.2022.08.001 PMC10508214

[37] J. M. H. Lau , The role of neuronal excitability, allocation to an engram and memory linking in the behavioral generation of a false memory in mice. Neurobiol. Learn. Mem. **174**, 107284 (2020). 32745601 10.1016/j.nlm.2020.107284 PMC7583262

[38] S. Williams, N. Mmbaga, S. Chirwa, Dopaminergic D1 receptor agonist SKF 38393 induces GAP-43 expression and long-term potentiation in hippocampus in vivo. Neurosci. Lett. **402**, 46–50 (2006). 16675111 10.1016/j.neulet.2006.03.075

[39] E. Messaoudi, S. W. Ying, T. Kanhema, S. D. Croll, C. R. Bramham, Brain-derived neurotrophic factor triggers transcription-dependent, late phase long-term potentiation in vivo. J. Neurosci. **22**, 7453–7461 (2002). 12196567 10.1523/JNEUROSCI.22-17-07453.2002 PMC6757978

[40] K. Wibrand , Identification of genes co-upregulated with Arc during BDNF-induced long-term potentiation in adult rat dentate gyrus in vivo. Eur. J. Neurosci. **23**, 1501–1511 (2006). 16553613 10.1111/j.1460-9568.2006.04687.x

[41] S. N. Yang, Sustained enhancement of AMPA receptor- and NMDA receptor-mediated currents induced by dopamine D1/D5 receptor activation in the hippocampus: An essential role of postsynaptic Ca2+ Hippocampus **10**, 57–63 (2000). 10706217 10.1002/(SICI)1098-1063(2000)10:1<57::AID-HIPO6>3.0.CO;2-0

[42] Y. Y. Huang, E. R. Kandel, D1/D5 receptor agonists induce a protein synthesis-dependent late potentiation in the CA1 region of the hippocampus. Proc. Natl. Acad. Sci. U.S.A. **92**, 2446–2450 (1995). 7708662 10.1073/pnas.92.7.2446 PMC42234

[43] S. Navakkode, S. Sajikumar, T. C. Sacktor, J. U. Frey, Protein kinase Mzeta is essential for the induction and maintenance of dopamine-induced long-term potentiation in apical CA1 dendrites. Learn. Mem. **17**, 605–611 (2010). 21084457 10.1101/lm.1991910 PMC2998336

[44] S. Navakkode, S. Sajikumar, M. Korte, T. W. Soong, Dopamine induces LTP differentially in apical and basal dendrites through BDNF and voltage-dependent calcium channels. Learn. Mem. **19**, 294–299 (2012). 22723051 10.1101/lm.026203.112

[45] M. S. Shetty, S. Sajikumar, Differential involvement of Ca(2+)/calmodulin-dependent protein kinases and mitogen-activated protein kinases in the dopamine D1/D5 receptor-mediated potentiation in hippocampal CA1 pyramidal neurons Neurobiol. Learn. Mem. **138**, 111–120 (2017). 27470093 10.1016/j.nlm.2016.07.020

[46] T. J. Bacon, A. E. Pickering, J. R. Mellor, Noradrenaline release from locus coeruleus terminals in the hippocampus enhances excitation-spike coupling in CA1 pyramidal neurons via beta-Adrenoceptors. Cereb. Cortex **30**, 6135–6151 (2020). 32607551 10.1093/cercor/bhaa159 PMC7609922

[47] M. A. L. Quinlan , Locus coeruleus optogenetic light activation induces long-term potentiation of perforant path population spike amplitude in rat dentate gyrus. Front. Syst. Neurosci. **12**, 67 (2018). 30687027 10.3389/fnsys.2018.00067 PMC6333706

[48] J. P. Johansen , Hebbian and neuromodulatory mechanisms interact to trigger associative memory formation. Proc. Natl. Acad. Sci. U.S.A. **111**, E5584–5592 (2014). 25489081 10.1073/pnas.1421304111 PMC4280619

[49] M. A. Sutton, E. M. Schuman, Local translational control in dendrites and its role in long-term synaptic plasticity. J. Neurobiol. **64**, 116–131 (2005). 15883999 10.1002/neu.20152

[50] J. A. Varela, S. J. Hirsch, D. Chapman, L. S. Leverich, R. W. Greene, D1/D5 modulation of synaptic NMDA receptor currents. J. Neurosci. **29**, 3109–3119 (2009). 19279248 10.1523/JNEUROSCI.4746-08.2009 PMC2684496

[51] J. Jones-Tabah, H. Mohammad, E. G. Paulus, P. B. S. Clarke, T. E. Hebert, The signaling and pharmacology of the dopamine D1 receptor. Front. Cell Neurosci. **15**, 806618 (2021). 35110997 10.3389/fncel.2021.806618 PMC8801442

[52] W. B. Smith, S. R. Starck, R. W. Roberts, E. M. Schuman, Dopaminergic stimulation of local protein synthesis enhances surface expression of GluR1 and synaptic transmission in hippocampal neurons. Neuron **45**, 765–779 (2005). 15748851 10.1016/j.neuron.2005.01.015

[53] O. David, I. Barrera, N. Gould, S. Gal-Ben-Ari, K. Rosenblum, D1 dopamine receptor activation induces neuronal eEF2 pathway-dependent protein synthesis. Front. Mol. Neurosci. **13**, 67 (2020). 32499677 10.3389/fnmol.2020.00067 PMC7242790

[54] C. Gao, X. Sun, M. E. Wolf, Activation of D1 dopamine receptors increases surface expression of AMPA receptors and facilitates their synaptic incorporation in cultured hippocampal neurons. J. Neurochem. **98**, 1664–1677 (2006). 16800848 10.1111/j.1471-4159.2006.03999.x

[55] C. Rozas , Methylphenidate amplifies long-term potentiation in rat hippocampus CA1 area involving the insertion of AMPA receptors by activation of beta-adrenergic and D1/D5 receptors. Neuropharmacology **99**, 15–27 (2015). 26165920 10.1016/j.neuropharm.2015.07.003

[56] P. Park , The role of calcium-permeable AMPARs in long-term potentiation at principal neurons in the rodent hippocampus. Front. Synaptic Neurosci. **10**, 42 (2018). 30524263 10.3389/fnsyn.2018.00042 PMC6262052

[57] P. Park , Further evidence that CP-AMPARs are critically involved in synaptic tag and capture at hippocampal CA1 synapses. Mol. Brain **14**, 26 (2021). 33526063 10.1186/s13041-021-00737-2 PMC7851922

[58] R. L. Redondo , Synaptic tagging and capture: Differential role of distinct calcium/calmodulin kinases in protein synthesis-dependent long-term potentiation. J. Neurosci. **30**, 4981–4989 (2010). 20371818 10.1523/JNEUROSCI.3140-09.2010 PMC6632790

[59] P. V. Nguyen, J. N. Gelinas, Noradrenergic gating of long-lasting synaptic potentiation in the hippocampus: From neurobiology to translational biomedicine. J. Neurogenet. **32**, 171–182 (2018). 30175650 10.1080/01677063.2018.1497630

[60] N. Hansen, D. Manahan-Vaughan, Hippocampal long-term potentiation that is elicited by perforant path stimulation or that occurs in conjunction with spatial learning is tightly controlled by beta-adrenoreceptors and the locus coeruleus. Hippocampus **25**, 1285–1298 (2015). 25727388 10.1002/hipo.22436 PMC6680149

[61] T. Hatfield, J. L. McGaugh, Norepinephrine infused into the basolateral amygdala posttraining enhances retention in a spatial water maze task. Neurobiol. Learn Mem. **71**, 232–239 (1999). 10082642 10.1006/nlme.1998.3875

[62] S. J. Sara, C. Dyon-Laurent, A. Herve, Novelty seeking behavior in the rat is dependent upon the integrity of the noradrenergic system. Brain Res. Cogn. Brain Res. **2**, 181–187 (1995). 7580400 10.1016/0926-6410(95)90007-1

[63] L. Cahill, C. A. Pham, B. Setlow, Impaired memory consolidation in rats produced with beta-adrenergic blockade. Neurobiol. Learn. Mem. **74**, 259–266 (2000). 11031131 10.1006/nlme.1999.3950

[64] J. L. Swanson-Park , A double dissociation within the hippocampus of dopamine D1/D5 receptor and beta-adrenergic receptor contributions to the persistence of long-term potentiation. Neuroscience **92**, 485–497 (1999). 10408599 10.1016/s0306-4522(99)00010-x

[65] M. Shivarama Shetty, S. Gopinadhan, S. Sajikumar, Dopamine D1/D5 receptor signaling regulates synaptic cooperation and competition in hippocampal CA1 pyramidal neurons via sustained ERK1/2 activation. Hippocampus **26**, 137–150 (2016). 26194339 10.1002/hipo.22497 PMC5054950

[66] J. Lisman, K. Cooper, M. Sehgal, A. J. Silva, Memory formation depends on both synapse-specific modifications of synaptic strength and cell-specific increases in excitability. Nat. Neurosci. **21**, 309–314 (2018). 29434376 10.1038/s41593-018-0076-6 PMC5915620

[67] A. Chowdhury, P. Caroni, Time units for learning involving maintenance of system-wide cFos expression in neuronal assemblies. Nat. Commun. **9**, 4122 (2018). 30297716 10.1038/s41467-018-06516-3 PMC6175937

[68] E. I. Moser, K. A. Krobert, M. B. Moser, R. G. Morris, Impaired spatial learning after saturation of long-term potentiation. Science **281**, 2038–2042 (1998). 9748165 10.1126/science.281.5385.2038

[69] V. H. Brun, K. Ytterbo, R. G. Morris, M. B. Moser, E. I. Moser, Retrograde amnesia for spatial memory induced by NMDA receptor-mediated long-term potentiation. J. Neurosci. **21**, 356–362 (2001). 11150353 10.1523/JNEUROSCI.21-01-00356.2001 PMC6762446

[70] W. C. da Silva, C. C. Kohler, A. Radiske, M. Cammarota, D1/D5 dopamine receptors modulate spatial memory formation. Neurobiol. Learn. Mem. **97**, 271–275 (2012). 22266268 10.1016/j.nlm.2012.01.005

[71] C. Harley, Noradrenergic and locus coeruleus modulation of the perforant path-evoked potential in rat dentate gyrus supports a role for the locus coeruleus in attentional and memorial processes. Prog. Brain Res. **88**, 307–321 (1991). 1687619 10.1016/s0079-6123(08)63818-2

[72] G. Paxinos, C. Watson, The Rat Brain in Stereotaxic Coordinates (Academic Press, London, 2007).

