# Supplementary material for: Cell-type-specific optogenetic stimulation of the locus coeruleus induces slow-onset potentiation and enhances everyday memory in rats
Source: Proc Natl Acad Sci U S A. 2023 Nov 6;120(46):e2307275120. doi: 10.1073/pnas.2307275120 (PMC10655220; doi:10.1073/pnas.2307275120)
Supplement: Supplementary file 1 — Appendix 01 (PDF) [file pnas.2307275120.sapp.pdf]

Supporting Information for

Cell-type specific optogenetic stimulation of the locus coeruleus induces slow-onset potentiation and enhances everyday memory in rats.

Dorothy Tse*[1,2], Lucia Privitera*[1,3,4], Anna C Norton[1], Francesco Gobbo[1], Patrick Spooner[1], Tomonori Takeuchi[5,6], Stephen J Martin†[3] & Richard G M Morris†[1]
* equal contribution

† Corresponding authors
[1] Centre for Discovery Brain Sciences, Edinburgh Neuroscience, University of Edinburgh, United Kingdom
[2] Department of Psychology, Edge Hill University, United Kingdom
[3] School of Medicine, University of Dundee, United Kingdom
[4] Barts and the London School of Medicine, Queen Mary University of London Malta Campus, Malta
[5] Danish Research Institute of Translational Neuroscience - DANDRITE, Nordic-EMBL Partnership for Molecular Medicine, Aarhus University, Denmark
[6] Center for Proteins in Memory - PROMEMO, Danish National Research Foundation, Department of Biomedicine, Aarhus University, Denmark

Corresponding Author Email: r.g.m.morris@ed.ac.uk

This PDF file includes:

Figures S1 to S6
Legends for figures S1 to S6

Other supporting materials for this manuscript include the following:

Dataset S1

Supplementary Methods

Viral Vectors

Cre-inducible AAVs were obtained from the University of Zurich Viral Vector Facility. The viral titer was 4.9×10^{12} vg/ml for ssAAV-DJ/2-hSyn1-dlox-ChrimsonR-tdTomato (rev)-dlox-WPRE-bGHp(A) (v289-DJ, ChR^{+}) and 5.5×10^{12} vg/ml for ssAAV-DJ/2-hSyn1-dlox-tdTomato (rev)-dlox-WPRE-bGHp(A) (v284-DJ, ChR^{-}). The virus was divided into aliquots and stored at −80 °C until use.

In vivo optrode recordings

Three to four weeks before the optrode recordings Th-Cre rats were injected bilaterally in the LC with the Cre-inducible AAV-DJ ChR^{+} virus (ChR^{+}, $n = 3$). Once the injection was completed and the syringe removed from the brain, cerebrospinal fluid (CSF) liquid and blood residue were absorbed with sterile paper and the holes in the skull were covered with a sterile sponge. On the day of the optrode recording the rat was anesthetized with urethane [ethyl carbamate (Sigma-Aldrich), 1.5g/kg of body weight, intraperitoneal (IP) injection] and placed in a stereotaxic frame, the skull was exposed and the sponge was removed. A parylene-coated tungsten microelectrode (diameter = 127 µm, AC resistance = 1.0 MΩ) was glued to a custom SMA optical patch cable (optic fiber diameter = 400 µM, numerical aperture = 0.5; Thorlabs): the microelectrode protruded 100 µm from the end of the fiber, and the whole assembly is referred to as an ‘optrode’. Before each experiment the integrity of the optic fiber was checked and the LED driver calibrated to evoke consistent power across experiments using a Slim Photodiode Power Sensor (Thorlabs). The optrode was then placed in a stereotaxic arm with 20-degree angle in the AP direction. From a starting position 3.1 mm posterior and 1.2 mm lateral to lambda at the dura surface, the optrode was positioned above the LC (DV, –2.8 mm), then gradually lowered in 50 µm increments till –6.6 mm. Multi-unit spiking activity was observed in the continuous wide-band local field potential

(LFP) sampled and recorded via a USB-6003 acquisition device (National Instruments) connected to a PC. Light stimulation at power levels ranging from 3 to 13 mW was delivered using a Fiber-Coupled LED (M625F2 - 625 nm, 13.2 mW (min), 1000 mA, SMA; Thorlabs). Optical stimulation and multi-unit recording were controlled by OG2, a custom-written LabVIEW program (National Instruments) developed by P.S.

In-vivo electrophysiology combined with optogenetic stimulation

Three to four weeks prior the electrophysiological recordings, Th-Cre rats were injected with either Cre-inducible AAV-DJ ChR^+ or ChR^- virus bilaterally in the LC as described above. Once the injection was completed and the syringe removed from the brain, cerebrospinal fluid (CSF) and blood residue were absorbed with sterile paper and the holes in the skull were covered with sterile sponge. After the surgery Rimadyl (carprofen, 0.08 ml/kg of body weight) was administered by subcutaneous injection. All rats were monitored for 7 days for them to regain their pre-surgery weights.

On the day of the electrophysiological recording rats were anaesthetized with urethane 1.5 g/kg (as described above) and placed in a stereotaxic frame with the skull horizontal. Body temperature was monitored by a rectal probe and maintained at 36.2 °C using a homeothermic heating blanket. Depth of anesthesia was assessed throughout the experiment, and urethane top-ups of 0.2 ml were administered as required. Subcutaneous injections of a glucose/saline mixture were administered every 3 h to maintain hydration (1.5 ml of 0.9% saline + 0.5 ml of 5% glucose). A fiber-optic cannula (MFC_400/430-0.48 NA_9mm_SM3_FLT Fiber-optic Cannula, Doric lenses) was screwed to a fiber-optic patch cord (MFP_400/460/ARMO-0.48_0.8m_SMA-CM3 Mono Fiber-optic Patch Cord, Doric lenses) and all connected to a Fiber-Coupled LED (M625F2 - 625 nm) controlled by an optic driver (LEDD1B). Prior the acute implantation of the fiber-optic cannulae a power test was performed to ensure the integrity of both the cannulae and the patchcord and to calibrate the optic driver to deliver 10 mW. The sponges previously placed in the skull during viral injection were removed to allow acute implantation of fiber-optic cannula above the LC (unilateral). A small burr-hole was made in the skull above the dorsal hippocampus, and a polytetrafluoroethylene-insulated monopolar platinum/iridium recording electrode (external diameter = 0.103 mm) was lowered into the stratum radiatum of area CA1 (coordinates from bregma: AP, –3.8 mm; ML, –2.5 mm; DV from the dura, ~–2.5 mm). A bipolar stimulating electrode comprising two twisted wires identical in composition to the recording electrode

was lowered into CA3 (coordinates from bregma: AP, –3.5 mm; ML, –3.0 mm; DV from the dura, ~−3.0 mm) in order to activate the Schaffer collateral input to CA1. Recordings were referenced to a skull screw positioned on the left parietal bone.

Stimulation and recording were controlled by a PC running custom-written LabView software (Evoked Potential Sampler, P.S.) for the control of electrical stimulation and the time-locked recording and online analysis of evoked field excitatory postsynaptic potentials (fEPSPs). Stimulation comprised biphasic constant-current pulses delivered via a pair of stimulus isolators (NL800A, Digitimer). fEPSPs were amplified and filtered (high pass = 1 Hz, low pass = 5 kHz) using a differential AC amplifier (Model 1700, A-M Systems), and sampled at 20 kHz using a PC data acquisition card (PCIe-6321, National Instruments). The slope of the early rising phase of each fEPSP (measured by linear regression between two fixed time points) and the peak amplitude were recorded.

At the start of each experiment, electrodes were lowered into the hippocampus: correct placement was determined based on the characteristic depth profiles observed during implantation (10), and final depths were adjusted to maximize the amplitude of the negative-going apical dendritic fEPSP elicited in CA1 by stimulation of CA3. Stimulation current was adjusted to evoke a fEPSP with a slope value of circa 50% of the maximum value obtained during an initial input-output curve.

After electrode placement, the fiber-optic cannula was lowered above the ipsilateral LC (identical coordinates to those described above). Baseline recording then began; single biphasic test pulses (50-µs pulse width per phase) were delivered at 2-min intervals. After recording a 1-h stable baseline, optical stimulation was delivered to LC-TH^+ neurons, comprising 20 pulses at 25 Hz delivered every 5 s for a total duration of 300 s, i.e. 60 trains in total. Recording continued for at least 3 h after optical stimulation. fEPSP slope data were normalized to the mean of the 1-h baseline period (assigned a value of 100%), and group means were calculated.

Drugs associated with in-vivo electrophysiology

All drugs were dissolved in sterile saline and stored in aliquots at –20 °C before use. Concentrations and doses were as follows: SCH 23390 hydrochloride (Tocris Bioscience), 1 mg/kg of body weight; (S)-(−)-propranolol hydrochloride (Sigma-Aldrich 6.25 mg/kg; (±)-SKF 38393 (Sigma-Aldrich), 3 mg/kg. All drugs were administered by IP injection. SCH

23390 or propranolol were administered 30 min before the onset of light stimulation, and SKF 38393 was injected instead of light stimulation in WT rats.

Histology

Rats were terminally anaesthetised with sodium pentobarbital (Euthatal, 100 mg/kg of body weight, IP) and perfused transcardially with 4% paraformaldehyde (PFA) dissolved in phosphate buffered saline (PBS), pH 7.2. Brains were removed and stored in 4% PFA overnight at 4 °C, transferred to 30% sucrose in PBS for 2 days and then kept at –80 °C. Coronal sections (60 μm) were cut using a cryostat for histological analysis.

To check the position of each bilateral drug cannula in the dorsal hippocampus (n = 10), sections were then mounted on slides, stained with cresyl violet and coverslipped using mounting media DPX. The sections were examined using a light microscope under 20-fold magnification to verify the drug cannula placements. These locations were then marked on the appropriate coronal sections from the Paxinos and Watson (2007) brain atlas (72).

To check the viral expression and optic cannula position in the LC (n = 14), coronal sections were washed in PBS, permeabilised with 10% normal donkey serum (Sigma-Aldrich) and 0.5% Triton X-100 in PBS. They were then incubated overnight at room temperature with a monoclonal mouse anti-TH antibody (1:1000 dilution; 22941, Immunostar) and a polyclonal rabbit anti-RFP antibody (1:200 dilution; ab62341, Abcam). Sections were then washed and incubated with secondary antibodies (1:200 dilution; donkey anti-mouse 647 and donkey anti-rabbit 488). After washing, slices were mounted in Fluoroshield with DAPI (Sigma-Aldrich). The deepest optic cannula position in the LC on the stained sections was located under a fluorescent microscope with 20-fold magnification and measured using Image-Pro (Media Cybernetics).

To confirm anterograde projections from the LC to the dorsal hippocampus, coronal slices from Th-Cre rats (n = 5) injected with Cre-inducible AAV-DJ ChR^+ virus in the LC were stained with mouse anti-NET (1:1000 dilution; ARG11072, arigo Biolaboratories) and rabbit anti-TH (1:1000 dilution, AB152, Millipore), followed by secondary antibodies donkey anti-mouse-Alexa 647 (1:200 dilution) and donkey anti-rabbit-Alexa 488 (1:200 dilution). These coronal slices were also stained with rabbit anti-RFP (1:200 dilution; ab6234, Abcam) and mouse anti-Synaptophysin 1 (SYP1) (1:500 dilution; 101 011, Synaptic Systems), followed by anti-rabbit-Alexa 488 (1:200 dilution) and anti-mouse-Alexa 647 (1:200 dilution).

All images were acquired with Nikon A1R (Nikon) or LSM800 (ZEISS) and 10× (CFI Plan Fluor 10×/0.30 DIC L/N1, Nikon), 20× (CFI Plan Apo VC 20×/0.75 DIC N2, Nikon or Plan-Apochromat 20×/0.8 M27, ZEISS) objectives.

Statistical analysis

Statistical analyses were performed using IMB SPSS Statistics 28 (IMB) and Prism (GraphPad). All data were analyzed by ANOVA followed, where appropriate, by post-hoc pairwise comparisons (Tukey’s HSD), or by independent-sample, paired-sample, or one-sample t tests as appropriate. Data are reported as mean ± SEM.

Figure S1 Total Dig time

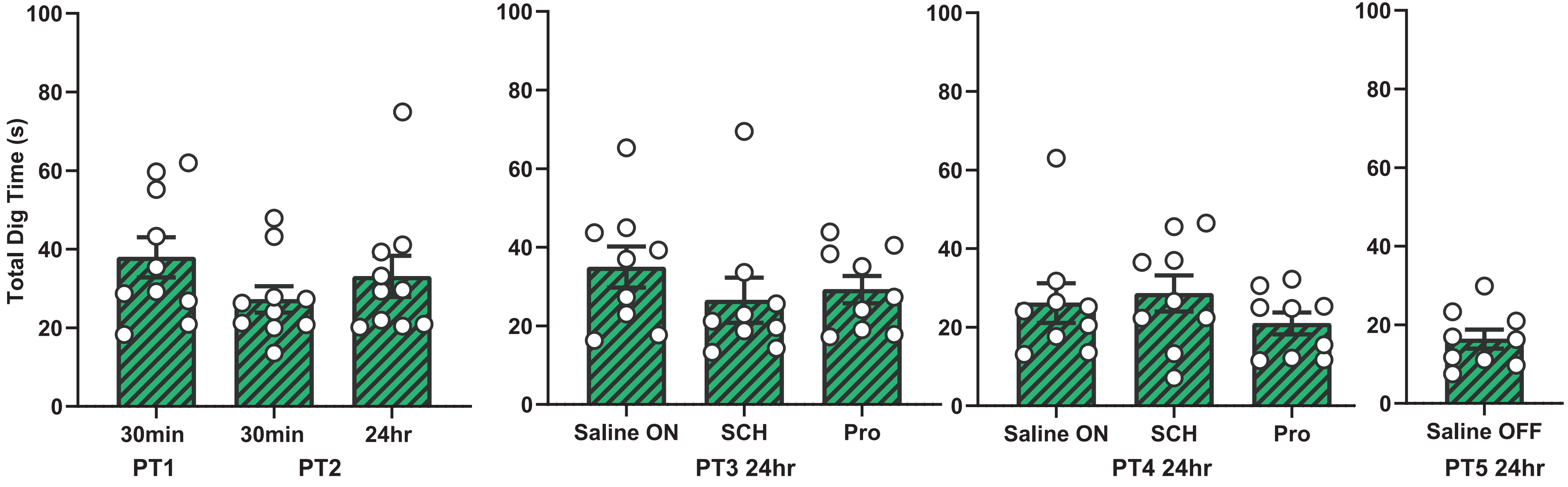

Figures S1 *Total dig time across all probe trials (PTs)*. The absolute total dig time was c. 30 - 40 s in early PTs (1-3). There was a trend towards slightly less digging in the correct location on later probe trials (PT 4-5). However, there were no significant differences between conditions in any of the PTs (F < 1, n.s).

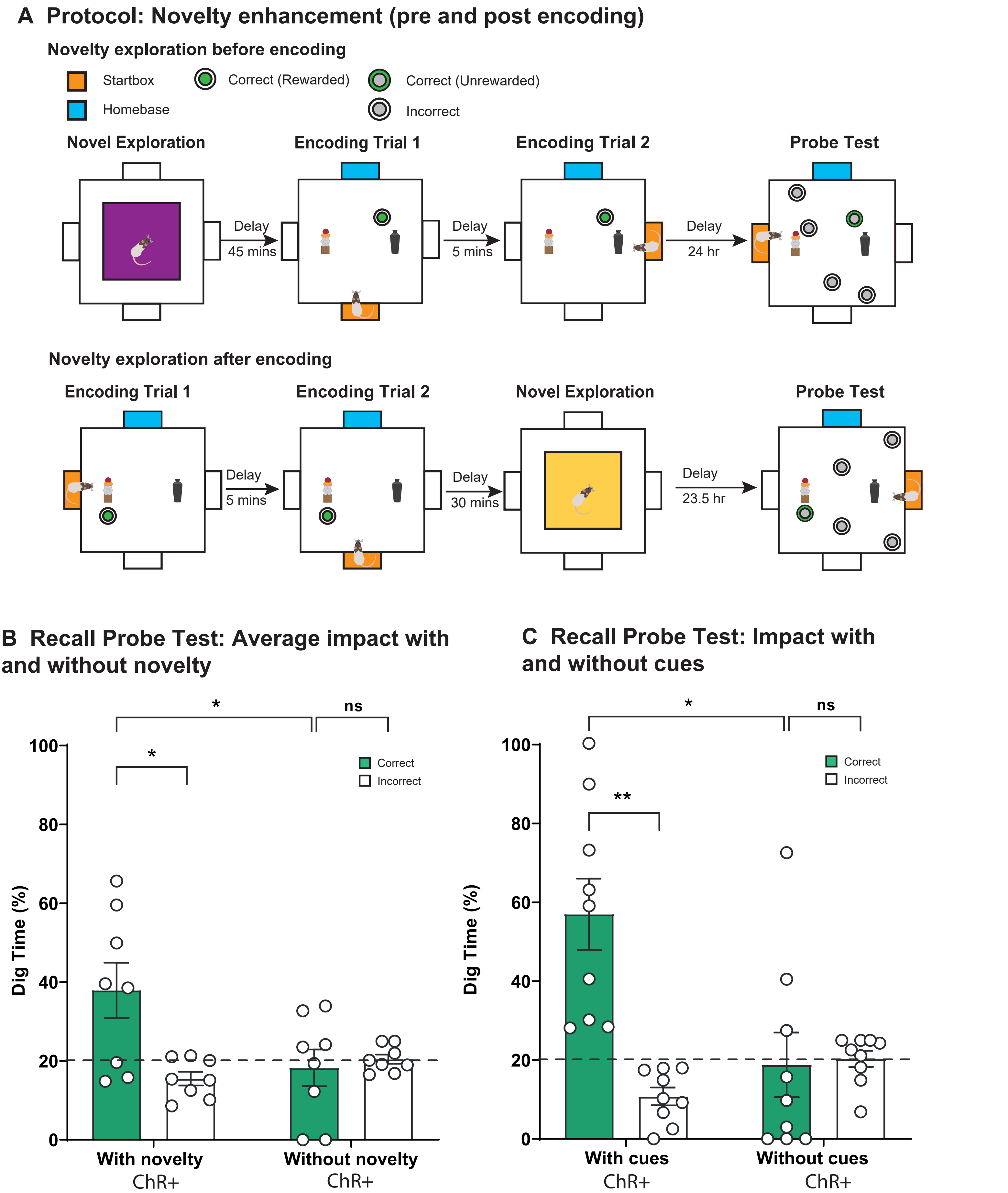

Figures S2 *Novelty exploration and removal of intramaze and extramaze cues*. (A) Experimental design for novelty before and after encoding. (B) Memory retention was enhanced by environmental novelty at 24 hr interval. (C) In a recall PT at 30 min memory retention was very good when cues were present (>50 %) but fell to chance levels when cues were absent. Means ± 1 SEM and individual animal data plots.

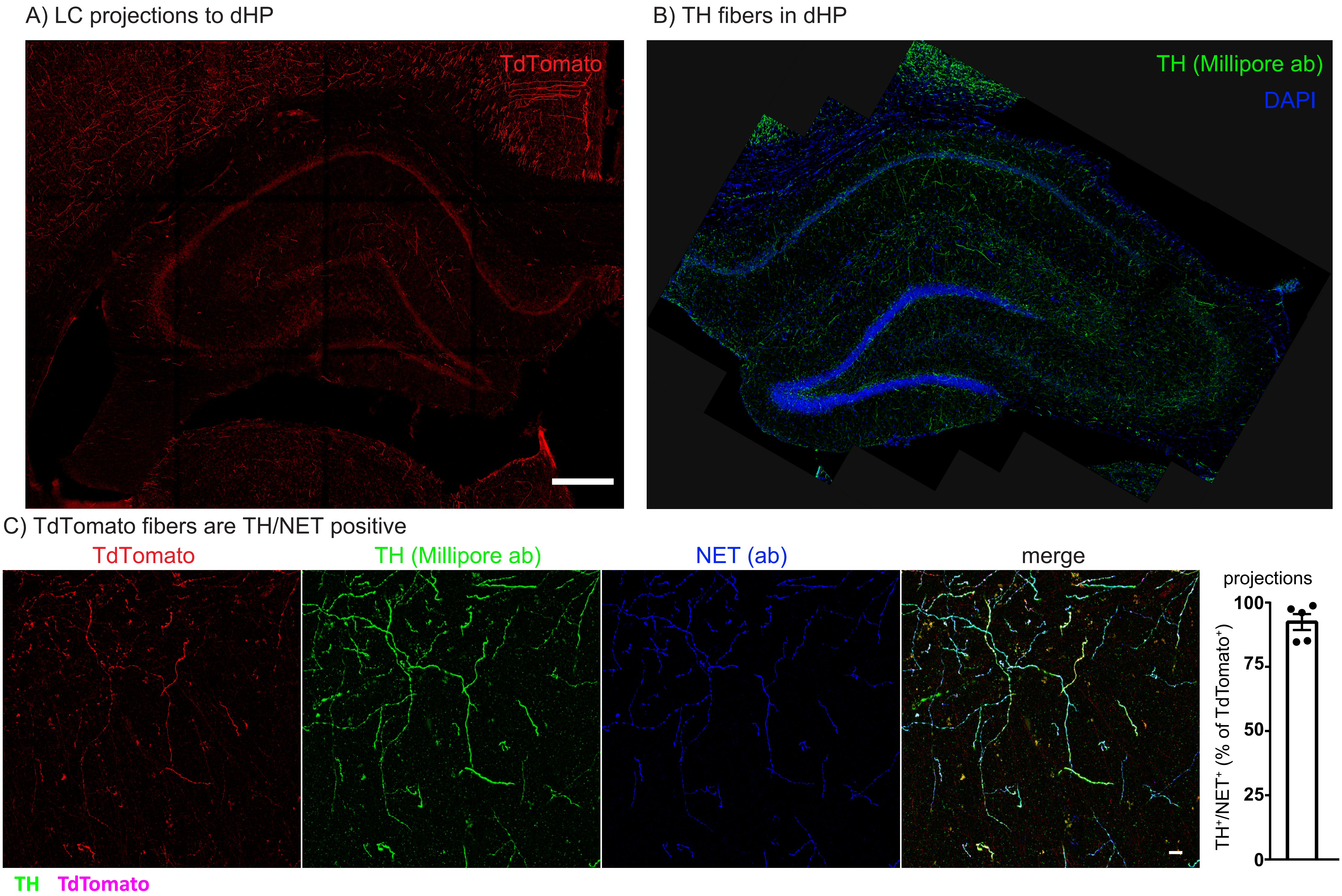

Figure S3. *LC projections to dorsal hippocampus.* (A) Representative image of TdTomato+ positive fibers in the dorsal hippocampus (red). The image is representative of 4 animals analysed. Scale bar, 100µm. (B) Representative image of TH projections to the dorsal hippocampus stained with the anti-TH Millipore antibody (see Methods) (green). Nuclei are stained with DAPI (blue). The image is representative of 3 animals analysed. (C) immunostaining of hippocampal sliced from animals expressing TdTomato in LC neurons. From left to right, in red anti-RFP (TdTomato) signal, in green anti-TH staining, in blue anti-NET staining, and merge. Scale bar, 50µm. On the right, quantification of the fraction of double positive TH/NET fibers out of the total TdTomato+ fibers, n=5 animals.

Figure S4

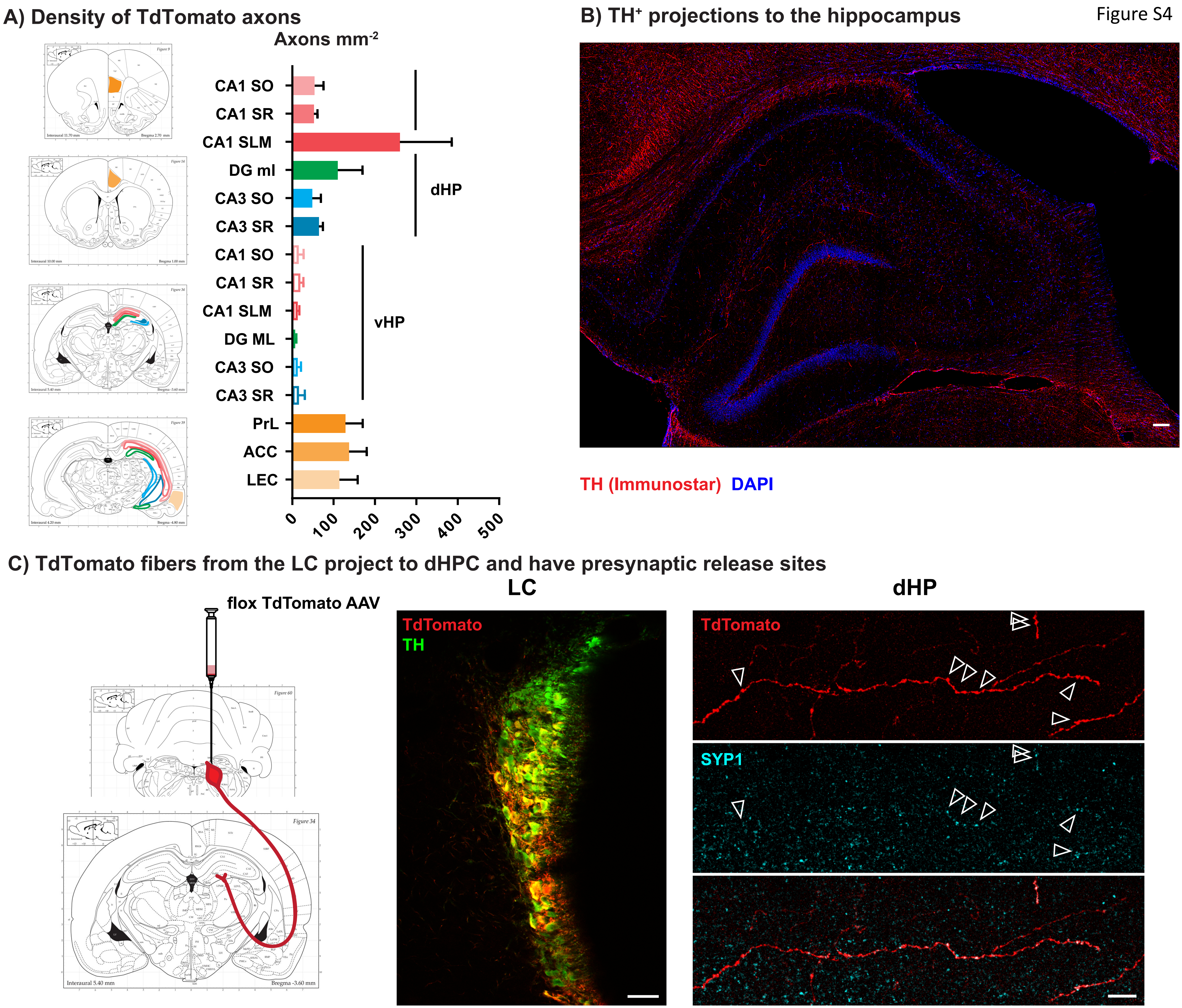

Figure. S4 *TH projections to dorsal hippocampus.* (A) Quantification of number of TH+ positive fibers in cortical and hippocampal subregions. dHP : dorsal hippocampus, vHP: ventral hippocampus, SO: stratum oriens, SR: stratum radiatum, SLM: stratum lacunosum-molecolare, ML: molecular layer, PrL: prelimbic cortex, ACC: anterior cingulate cortex, LEC: lateral entorhinal cortex. (B) Typical image used in the quantification, scale bar 100µm. (C) LC neurons have synaptic terminals in dorsal hippocampus. Terminals from LC neurons expressing TdTomato were identified in dorsal hippocampal slices and stained for presynaptic marker SYP1. Representative images of 3 animals analysed. Left panel, schematic of experiment, middle panel, LC coronal slice stained for TH (green) scale bar 50µm, right panel hippocampal slice stained for SYP1 (cyan). Arrowheads indicate presynaptic terminals on TdTomato+ axons, scale bar 10µm.

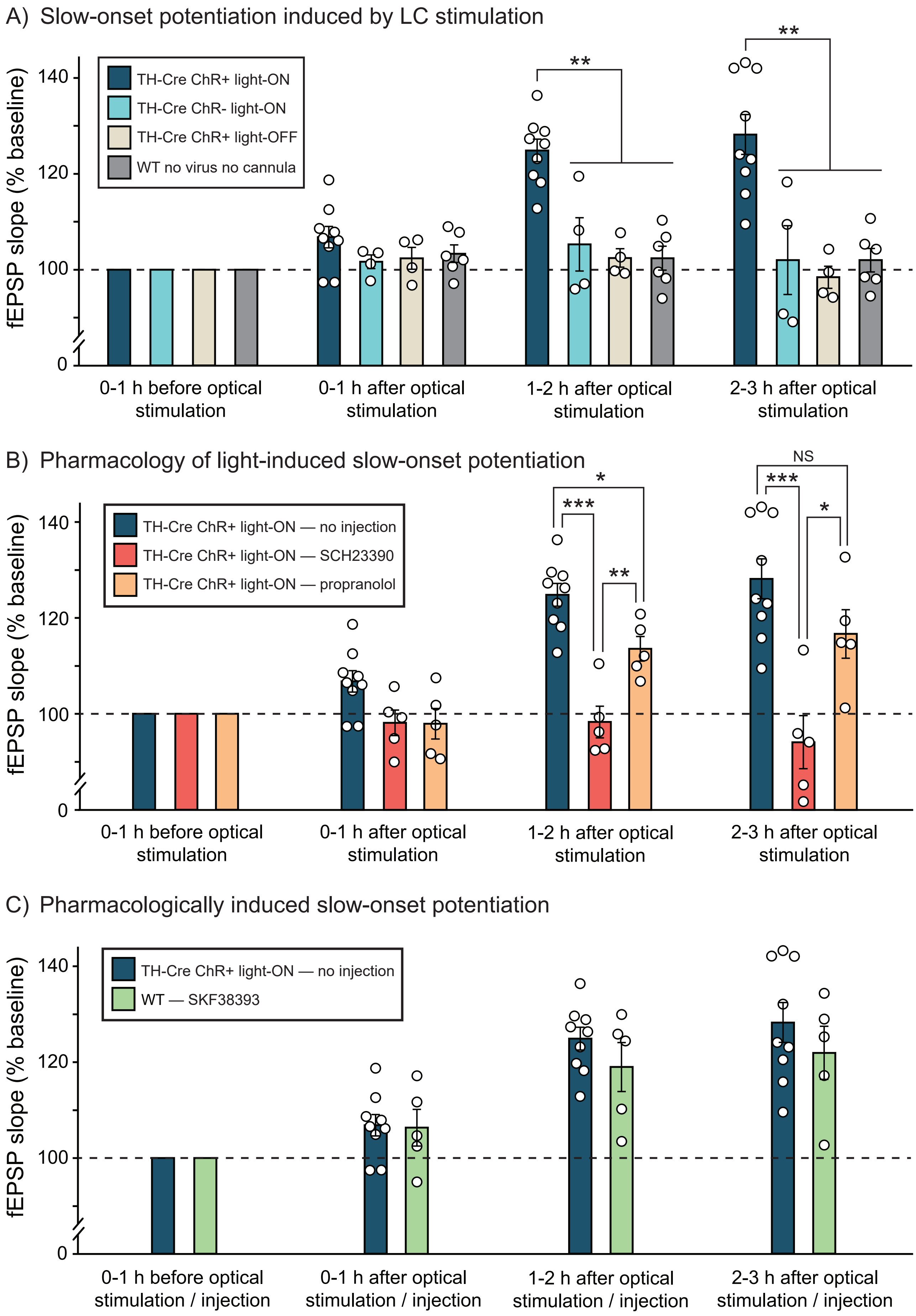
A) Slow-onset potentiation induced by LC stimulation
TH-Cre ChR+ light-ON
TH-Cre ChR- light-ON
TH-Cre ChR+ light-OFF
WT no virus no cannula
fEPSP slope (% baseline)
140
120
100
0
**
**
0-1 h before optical stimulation
0-1 h after optical stimulation
1-2 h after optical stimulation
2-3 h after optical stimulation
B) Pharmacology of light-induced slow-onset potentiation
TH-Cre ChR+ light-ON — no injection
TH-Cre ChR+ light-ON — SCH23390
TH-Cre ChR+ light-ON — propranolol
fEPSP slope (% baseline)
140
120
100
0
*
***
**
NS
***
*
0-1 h before optical stimulation
0-1 h after optical stimulation
1-2 h after optical stimulation
2-3 h after optical stimulation
C) Pharmacologically induced slow-onset potentiation
TH-Cre ChR+ light-ON — no injection
WT — SKF38393
fEPSP slope (% baseline)
140
120
100
0
0-1 h before optical stimulation / injection
0-1 h after optical stimulation / injection
1-2 h after optical stimulation / injection
2-3 h after optical stimulation / injection

Figure S5 *Slow-onset potentiation* The data was averaged over 1-h time periods relative to the onset of light stimulation and normalized to the 1-h baseline period: supplementary analysis of the data presented in Fig. 3. Note that the baseline 0-1 h before light stimulation is always 100% by definition. (A) Slow-onset potentiation induced in the TH-Cre ChR+ light-ON group (n = 9) relative to the three control groups (TH-Cre ChR- light-ON; n = 4, TH-Cre ChR+ light-OFF; n = 4, and WT with no virus and no cannula; n = 6). A repeated-measures ANOVA in which treatment group was entered as a between-subjects factor, and time after optical stimulation (0-1 h, 1-2 h, and 2-3 h) was entered as a within-subjects factor, revealed a significant main effect of group [F = 15.5, df = 3/19, p < 0.001] and a significant group x time interaction [F = 6.72, df = 6/38, p < 0.001]. Post-hoc pairwise comparisons with Bonferroni correction revealed significant differences between the TH-Cre ChR+ light-ON group and both other groups 1-2 h after light stimulation (***p < = 0.001 in all cases), and 2-3 h after stimulation (**p < 0.01; *** p < 0.001). Comparisons with baseline (100%) at each time point (one-sample t-tests with Bonferroni corrections for multiple comparisons) revealed no significant potentiation in any of the control groups; there was a non-significant trend toward potentiation in the TH-Cre ChR+ light-ON group 0-1 h after optical stimulation [t = 2.99, df = 8, p = 0.068], and potentiation was significant after 1-2 h [t = 10.5, df = 8, p < 0.001], and after 2-3 h [t = 6.79, df = 8, p < 0.001]. (B) The impact of SCH23390 (1 mg/kg; n = 5) and propranolol (6.25 mg/kg; n = 5) injection on light-induced slow-onset potentiation relative to the TH-Cre ChR+ light-ON ('no injection') group (n = 9). SCH23390 caused a complete block of slow-onset potentiation, whereas a delayed onset and partial blockade was observed after propranolol injection. An ANOVA revealed a significant main effect of group [F = 23.0; df = 2,16, p < 0.001], and a significant treatment group x time interaction [F = 4.67; df = 4/32, p = 0.004]. Post-hoc pairwise comparisons with Bonferroni correction revealed significant differences between all groups 1-2 h after optical stimulation (*p < 0.05; **p < 0.01; ***p < 0.001), and differences between the SCH23390 group and both other groups 2-3 h after stimulation (*p < 0.05; ***p < 0.001). However, the difference between the no injection group and the propranolol group was no longer significant 2-3 h after stimulation (p = 0.33). (C) Light-induced and pharmacologically induced slow-onset potentiation did not differ. An AVOVA revealed no difference between the no injection TH-Cre ChR+ light-ON group (n = 9) and the SKF38393-injected group (n = 5) [F = 1.06, df = 1/12, p = 0.33], but a highly significant overall increase was observed over time [F = 20.8, df = 2/24, p < 0.001]. Comparisons with baseline (100%) at each time point (both groups combined owing to the absence of a group difference; one-sample t-tests with Bonferroni corrections for multiple comparisons) revealed significant potentiation at all times [0-1 h: t = 3.44, df = 13, p = 0.008; 1-2 h: t = 9.52, df = 13, p < 0.001; 2-3 h: t = 7.85. df = 13, p < 0.001].

Figure S6

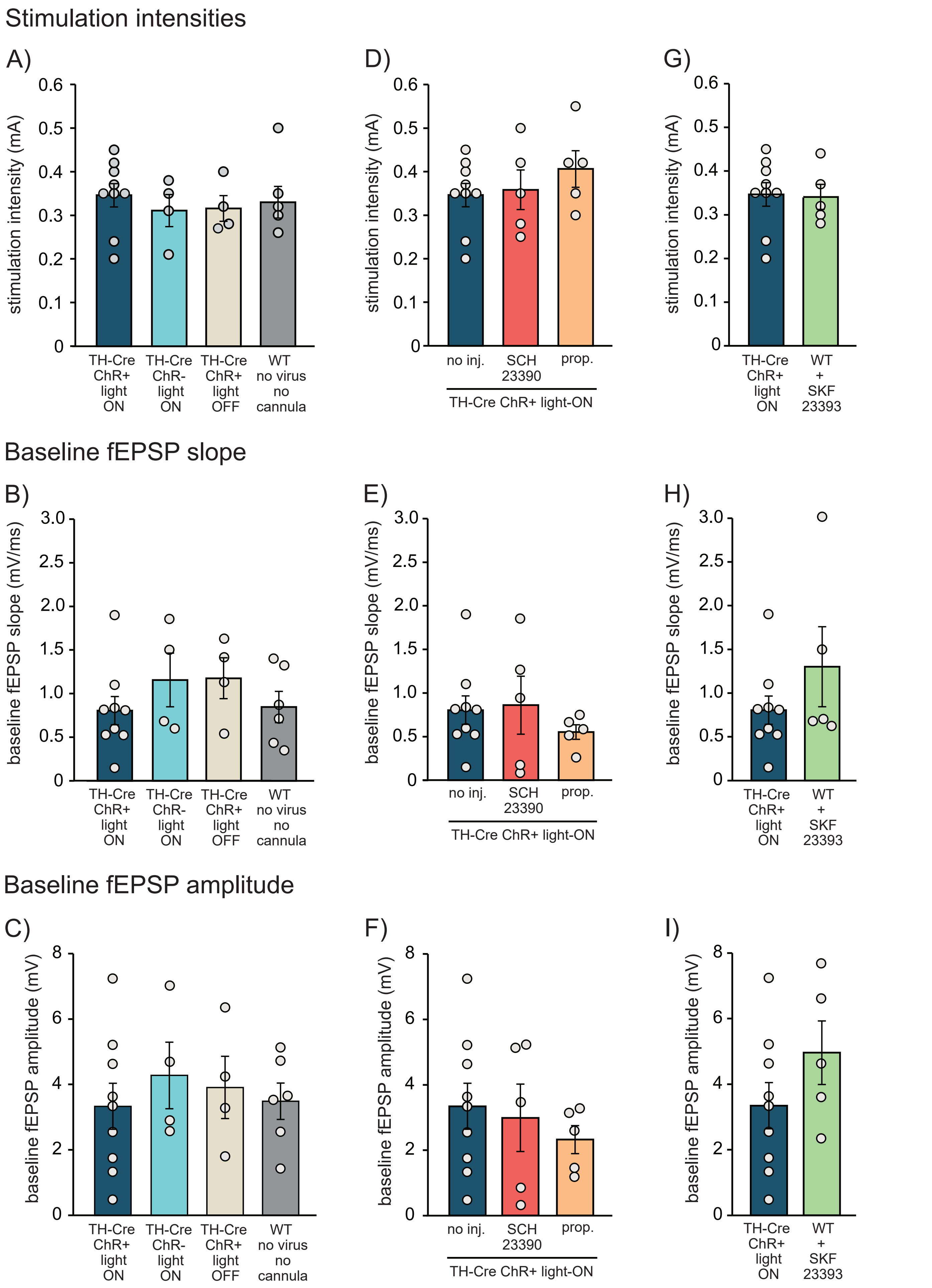
Stimulation intensities
A)
D)
G)
stimulation intensity (mA)
0.6
0.5
0.4
0.3
0.2
0.1
0
TH-Cre ChR+ light ON
TH-Cre ChR- light ON
TH-Cre ChR+ light OFF
WT no virus no cannula
no inj.
SCH 23390
prop.
TH-Cre ChR+ light-ON
WT + SKF 23393
Baseline fEPSP slope
B)
E)
H)
baseline fEPSP slope (mV/ms)
3.0
2.5
2.0
1.5
1.0
0.5
Baseline fEPSP amplitude
C)
F)
I)
baseline fEPSP amplitude (mV)
8
6
4
2

Figure S6. *Baseline parameters of electrophysiology.* (A) Stimulation intensities used to elicit a fEPSP with 50% maximal slope in the light-induced slow-onset potentiation group (TH-Cre, ChR+, light-ON) versus the 3 control groups (TH-Cre, ChR-, light-ON; TH-Cre, ChR+, light-OFF; WT, no virus, no cannula); there was no group difference ($F = 0.24$, $df = 3/19$, $p = 0.86$). (B) Mean baseline fEPSP slope over the 1-h baseline period in the light-induced slow-onset potentiation group versus the 3 control groups; there was no group difference ($F = 0.85$, $df = 3/19$, $p = 0.48$). (C) Baseline fEPSP amplitude in the light-induced slow-onset potentiation group versus the 3 control groups; there was no group difference ($F = 0.27$, $df = 3/19$, $p = 0.85$). (D) Stimulation intensities in the light-induced slow-onset potentiation group versus the groups in which light-stimulation was delivered in the presence of SCH23390 or propranolol; there was no group difference ($F = 0.74$, $df = 2/16$; $p = 0.49$). (E) Baseline fEPSP slope in the light-induced slow-onset potentiation group versus the groups in which light-stimulation was delivered in the presence of SCH23390 or propranolol; there was no group difference ($F = 0.53$, $df = 2/16$, $p = 0.60$).
(F) Baseline fEPSP amplitude in the light-induced slow-onset potentiation group versus the groups in which light-stimulation was delivered in the presence of SCH23390 or propranolol; there was no group difference ($F = 0.44$, $df = 2/16$, $p = 0.65$). (G) Stimulation intensities in the light- versus SKF38393-induced potentiation groups; there was no group difference ($t = 0.14$, $df = 12$, $p = 0.89$). (H) Baseline fEPSP slope in the light- versus SKF38393-induced potentiation groups; there was no group difference ($t = 1.25$, $df = 12$, $p = 0.24$). (I) Baseline fEPSP amplitude in the light- versus SKF38393-induced potentiation groups; there was no group difference ($t = 1.36$; $df = 12$, $p = 0.20$).
